# Expression of antibody fragments in *Saccharomyces cerevisiae* strains evolved for enhanced protein secretion

**DOI:** 10.1186/s12934-021-01624-0

**Published:** 2021-07-14

**Authors:** Yanyan Wang, Xiaowei Li, Xin Chen, Jens Nielsen, Dina Petranovic, Verena Siewers

**Affiliations:** 1 Department of Biology and Biological Engineering, Chalmers University of Technology, Gothenburg, Sweden; 2 Novo Nordisk Foundation Center for Biosustainability, Chalmers University of Technology, Gothenburg, Sweden; 3 Novo Nordisk Foundation Center for Biosustainability, Technical University of Denmark, Lyngby, Denmark

**Keywords:** Pharmaceutical proteins, *Saccharomyces cerevisiae*, Protein secretion capacity, RNA-seq analysis

## Abstract

Monoclonal antibodies, antibody fragments and fusion proteins derived thereof have revolutionized the practice of medicine. Major challenges faced by the biopharmaceutical industry are however high production costs, long processing times and low productivities associated with their production in mammalian cell lines. The yeast *Saccharomyces cerevisiae*, a well-characterized eukaryotic cell factory possessing the capacity of post­translational modifications, has been industrially exploited as a secretion host for production of a range of products, including pharmaceuticals. However, due to the incompatible surface glycosylation, few antibody molecules have been functionally expressed in *S. cerevisiae*. Here, three non-glycosylated antibody fragments from human and the Camelidae family were chosen for expression in a *S. cerevisiae* strain (HA) previously evolved for high α-amylase secretion. These included the Fab fragment Ranibizumab (Ran), the scFv peptide Pexelizumab (Pex), and a nanobody consisting of a single V-type domain (Nan). Both secretion and biological activities of the antibody fragments were confirmed. In addition, the secretion level of each protein was compared in the wild type (LA) and two evolved strains (HA and MA) with different secretory capacities. We found that the secretion of Ran and Nan was positively correlated with the strains’ secretory capacity, while Pex was most efficiently secreted in the parental strain. To investigate the mechanisms for different secretion abilities in these selected yeast strains for the different antibody fragments, RNA-seq analysis was performed. The results showed that several bioprocesses were significantly enriched for differentially expressed genes when comparing the enriched terms between HA.Nan vs. LA.Nan and HA.Pex vs. LA.Pex, including amino acid metabolism, protein synthesis, cell cycle and others, which indicates that there are unique physiological needs for each antibody fragment secretion.

**Supplementary Information:**

The online version contains supplementary material available at 10.1186/s12934-021-01624-0.

## Background

Production of monoclonal antibodies (mAbs) has over the last three decades evolved into an extremely important branch of the biopharmaceutical industry. The applications of mAbs are rapidly expanding, including protein-based in vitro diagnostic tests, high-affinity protein purification methods, and the treatment of a wide array of major human diseases such as cancer, inflammation, autoimmune disorders, as well as cardiovascular and infectious diseases [1–3]. More recently, interest has shifted towards the study of minimal antibody-binding fragments, owing to their prominent advantages, such as high specificity, higher affinity and tissue penetrability, superior stability and solubility, reduced immunogenicity as well as easier and less expensive large-scale production [1, 4]. Three classes of antibody fragments, including Fab (antigen-binding fragment), scFv (single-chain variable fragment), and the single V-type domain, represent successive waves of antibody fragment technologies [5, 6]. However, isolation of intact and biologically specific antibody fragments by proteolysis such as papain and pepsin digestion is challenging [5, 7]. On the other hand, recombinant DNA technology opened the possibility of expressing antibody fragment genes in heterologous host organisms that are easy to manipulate and cultivate.

Mammalian cells and microorganisms, such as the bacterium *Escherichia coli* and yeasts, have been exploited as the main cell factories, which collectively account for the production of 89% of approved biopharmaceuticals [8]. Modified Chinese hamster ovary (CHO) cells produced up to 100 mg/L of VhHs in shake flasks [9]. The use of mammalian cells in bioprocessing, however, has some drawbacks, such as low product yield and growth rate, a high risk of viral contamination and the requirement for expensive growth medium. The use of microbial host organisms has therefore drawn increasing attention [8, 10]. Indeed, there have been several reports on engineering *E. coli* for the production of antibody fragments [7, 11, 12]. For example, through engineering of *E. coli*, several grams per liter of the recombinant antibody fragments scFv and Fab can be achieved in fermenters [13]. Compared with the bacterial expression systems, yeasts prove to be more desirable hosts for industrial-scale production of recombinant proteins due to their ability to perform post-translational modifications. Furthermore, their high robustness and tolerance towards harsh fermentation conditions, which are key factors in bioprocessing and scale-up, are additional advantages. Two anti-MUC1 VhHs were successfully expressed in *Pichia pastoris* for the first time, and titers in the range of 10–15 mg/L were obtained after a series of optimizations [14]. A major limitation for using *S. cerevisiae* as the antibody expression host is its native hyper-mannose glycosylation, which is highly antigenic in mammals [10, 15]. However, the absence of glycosylation sites on many antibody fragments circumvents this problem, further adding to the good prospect for yeast as a platform for production of this group of pharmaceuticals.

**Fig. 1:**
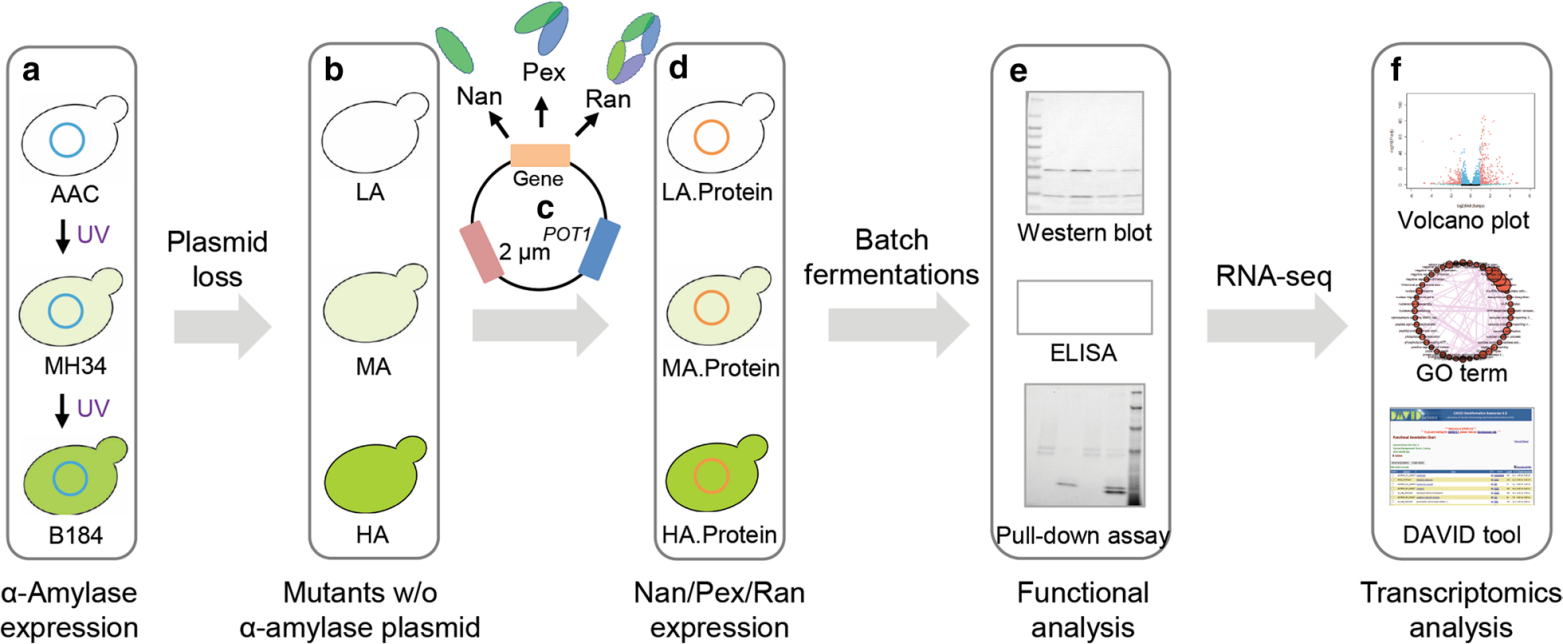
Schematic workflow for exploring the secretion capacity for antibody fragments of different mutant strains. **a** Three yeast strains evolved for significantly improved protein secretion based on α-amylase production. AAC, parental strain; MH34, intermediate strain; B184, highest level α-amylase secreting strain. **b** Strains after elimination of α-amylase plasmid from the mutant strains. LA, derived from AAC; MA, derived from MH34; HA, derived from B184. **c** Three new plasmids generated by inserting the three different antibody fragments genes (Nan, Pex and Ran) into the CPOTud vector, respectively. **d** Strains harboring plasmids for expression of Nan, Pex and Ran, respectively. Nine strains (LA.Nan, LA.Pex, LA.Ran, MA.Nan, MA.Pex, MA.Ran, HA.Nan, HA.Pex, and HA.Ran) were thus generated. **e** A series of functional analysis experiments was performed after shake flask fermentations of the strains harboring the different expression plasmids. **f** RNA-seq data were collected and integrated, resulting in an overall biological interpretation of antibody fragment production

*S. cerevisiae* is a preferred microbial cell factory for production of a variety of biopharmaceuticals due to its ability to secrete proteins, which facilitates their subsequent isolation, purification, and avoids the possible toxic intracellular build-up of foreign protein, and therefore significantly reduces the production costs [16, 17]. Although the secretory pathway in yeast is complex, it is still an attractive engineering target to improve the cell´s capacity to process the proteins of interest. Much work has been focused on overcoming the various limitations, optimizing the secretory processes [18, 19] and thus enhancing protein production. For instance, different antibodies and antibody fragments were successfully expressed in yeast [20–23], and engineering protein folding increased the antibody yields up to 10-fold [24]. Similar strategies were also employed to produce other proteins [21, 25] such as engineering vesicle trafficking, which improved the α-amylase titer up to 15-fold in glucose-limited fed batch cultivations [26]. Despite the success of targeted engineering, this has often been beneficial only for the production of the protein of interest, but not heterologous proteins in general [27]. Our laboratory previously isolated a group of yeast mutant strains with significantly elevated α-amylase production through random mutagenesis and microfluidic screening [28]. Among these strains, the best producer B184 was the descendant of MH34, which was in turn derived from AAC (the parental strain), and the production capacity gradually increased with each round of mutagenesis (Fig. 1) [28]. Furthermore, these mutant strains also showed increased secretion capacity for the other heterologous proteins, *Rhizopus oryzae* glucan 1,4-α-glucosidase [28] and *Trichoderma reesei* endo-1,4-beta-xylanase II [29]. Regardless of the progress in understanding protein secretion mechanisms, it is still not known whether the improved secretory capacity of these strains extends to heterologous proteins of pharmaceutical relevance, such as antibodies and antibody fragments.

Here, we characterized the expression of three representative antibody fragment drugs, including Ran (Ranibizumab, Fab fragment), Pex (Pexelizumab, scFv peptide) and Nan (Nanobody, single V-type domain) (Table 1), in a previously constructed high amylase productive strain HA (derived from B184). Subsequently, we demonstrated the biological activities of these proteins through ELISA and pull-down assay. Furthermore, we compared the production profiles of antibody fragments in different host variants LA (derived from AAC), MA (derived from MH34) and HA. Ultimately, RNA-seq was performed to explore the underlying mechanism upon observed differences in secretion capacities.

**Table 1:** Therapeutic antibody fragments used in this study

Generic name	Type	Structure^a^	Size	Isoelectric point	Disulfide bonds	Target	Indication
Nanobody (Nan, Camelid)	VhH	Single monomeric V_H_	17 kDa	5.92	2	Lysozyme	Rheumatoid arthritis and Crohn´s disease [30]
Pexelizumab (Pex, Humanized)	scFv	V_H_ and V_L_ connected by a polypeptide linker^b^	31 kDa	8.62	Uncertain	Complement C5	Coronary artery bypass and Angioplasty [31]
Ranibizumab (Ran, Humanized)	Fab	V_H_, V_L_, C_H_1 and C_L_	51 kDa	6.99	5	VEGF-A	Macular degeneration [32]

^a^V_H_, variable domain of heavy chain; V_L_, variable domain of heavy chain; C_H_1, the first constant domain of heavy chain; C_L_, constant domain of light chain

^b^The sequence of polypeptide linker is GGGGSGGGGSGGGGS

## Results

### **Secretion of antibody fragments by*S. cerevisiae***

**Fig. 2:**
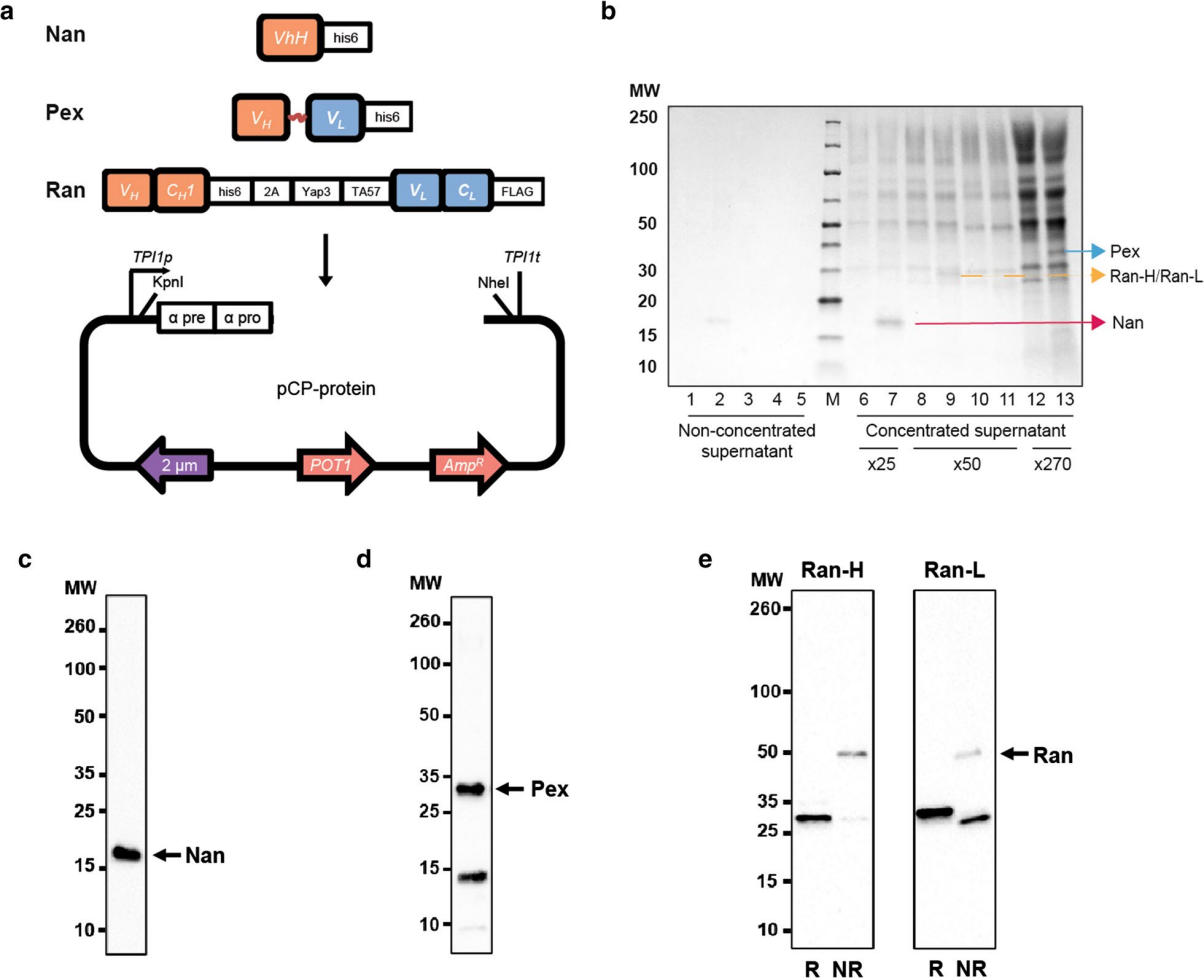
The expression of antibody fragments in *S. cerevisiae*. **a** Schematic of CPOTud-based vector for expression of antibody fragments in *S. cerevisiae*. VhH/V_H_, variable heavy chain; V_L_, variable light chain; C_H_1, the first constant domain of the heavy chain; C_L_, constant light chain; FLAG, FLAG-tag at the C-terminus of genes; his6, 6xHis-tag at the C terminus of genes; α-pre and α-pro, the components of α-factor leader, which is a native secretion leader; 2A, 2A peptide; Yap3-TA57, a synthetic secretion leader; *POT1*, selective marker from *Schizosaccharomyces pombe* to complement the lack of the *TPI1* gene in the host; 2 μm, 2 micron origin. **b** SDS-PAGE gel for the detection of antibody fragments in the HA host strain. 1, HA.CPOTud; 2, HA.Nan; 3, HA.Ran; 4, HA.Ran (non-reducing); 5, HA.Pex; M, protein ladder; 6, HA.CPOTud; 7, HA.Nan; 8, HA.CPOTud; 9, HA.Ran; 10, HA.CPOTud (non-reducing condition); 11, HA.Ran (non-reducing condition); 12, HA.CPOTud; 13, HA.Pex; 1–5, non-concentrated supernatant; 6–13, supernatants concentrated 25-, 50- or 270-fold using 10 K MW Pierce Concentrator PES. Arrows indicate expected proteins. MW, protein ladder. Western blots of cell supernatant from recombinant strains based on the HA host strain to detected proteins Nan (**c**), Pex (**d**) and Ran (**e**). Secreted antibody fragments were obtained after cultivation in SD-2xSCAA without BSA for 72 h. Nan, Pex and Ran-H were detected using an anti-6x-His-tag monoclonal antibody. Ran-L was detected using an anti-FLAG-tag monoclonal antibody. Nan and Pex were analyzed under reducing conditions. Ran was analyzed under reducing (R, left lane in each panel) and nonreducing (NR, right lane of each panel) conditions. The original images of the western blots are shown in Additional file 1: Fig. S1

In a previous study, B184 showed the highest α-amylase secretion level among all isolated mutant strains [28]. Therefore, we first expressed the antibody fragments in the HA host strain, i.e. B184 after the loss of the α-amylase expression plasmid. To maintain high plasmid stability and generate higher cell copy numbers, we used the CPOTud plasmid as the expression vector, in which the equivatent *POT1* gene from the glycolytic pathway of *Schizosaccharomyces pombe* was used as a selective marker to complement the lack of the *TPI1* gene in the host strain [33] (Fig. 2a). To direct heterologous proteins through the secretory pathway, the secretory signal peptide derived from the α-factor was added to the N-terminus of the antibody fragments. To ensure high translation initiation efficiency of the target proteins, a Kozak sequence (aacaaa) was inserted before the start codon of the gene [34]. In addition, a spacer sequence (EEGEPK) was included at the C-terminus of the leader peptide to increase the cleavage efficiencies of the pro-leader [35]. The resulting plasmids that encoded Nan (single V-type domain), Pex (scFv), and Ran (Fab) were introduced into HA, obtaining strains HA.Nan, HA.Pex and HA.Ran, respectively.

To confirm the expression and secretion of Nan and Pex proteins, we first performed an SDS-PAGE analysis (Fig. 2b). While showing a weak band for the Nan protein, no Pex was detected when the supernatant was not concentrated. After concentrating the supernatants of HA.Nan and HA.Pex 25-fold and 270-fold, the clear protein bands of 17 kDa and 31 kDa corresponding to Nan and Pex, respectively, were detected. Subsequently, we performed western blot analysis using an anti-6x-His-tag monoclonal antibody under reducing conditions. As shown in Fig. 2c and d (raw blots in Additional file 1: Fig. S1a and S1b), both detected protein signals were in agreement with the expected sizes. Interestingly, an additional protein band of ca. 14 kDa potentially stemming from partial degradation was found for the HA.Pex strain (Fig. 2d, Additional file 1: Fig. S1b), but this band was not detected in the control strain HA.CPOTud carrying the empty CPOTud vector. Previous studies reported that Kex2 endoprotease, specific for dibasic Lys-Arg sites, cleaves the leader-fusion protein in the late secretory pathway [35, 36]. As there is a Lys-Arg site present in the Pex protein sequence (K^129^R^130^), we hypothesized that the small band could be caused by Kex2 cleavage. To test this hypothesis, an R130K substitution was introduced into Pex, generating the strain HA.Pex (R130K). However, the continued appearance of this smaller fragment ruled out this possibility (Additional file 1: Fig. S2). We speculated that the additional band could result from a potential peptide bond hydrolysis that happens close to the linker sequence. The calculated molecular size might support this assumption.

Fab consists of two separate proteins including heavy chain and light chain. Therefore, the coordinated expression of these two chains is very important for the formation of the intact Fab. There are different possibilities to achieve this coordinated expression, e.g. the introduction of a 2A peptide sequence [37, 38]. Due to the polycistronic nature, small size and high “cleavage” efficiency, 2A peptides have received increased interest. 2A-peptide linked genes are translated from a single mRNA and “self-cleaved” through ribosomal skipping, which occurs co-translationally resulting in equal amounts of the co-expressed proteins [39]. In a previous study, the 2A peptide from Thosea asigna virus resulted in the highest cleavage efficiency and the highest expression level of three biosimilar IgG1 antibodies in CHO cells [40]. Therefore, it was considered interesting to evaluate expression of the third antibody fragment using this 2A self-processing peptide. We introduced the 2A peptide coding sequence into the Ran gene, an additional KR sequence and a GSG linker in front of the 2A peptide, an alpha factor leader and synthetic leader Yap3-TA57 at the N terminus of heavy chain (Ran-H) and light chain (Ran-L) and a 6xHis-tag and FLAG-tag at the C terminus of Ran-H and Ran-L, respectively. Subsequently, we characterized the expression of Ran-H and Ran-L using the 2A-containing construct. After concentrating the supernatant of HA.Ran 50-fold, we observed a band between 20 kDa and 30 kDa compared to concentrated HA.CPOTud under reducing conditions on the SDS-PAGE gel. But the similar molecular weights of Ran-H (26 kDa) and Ran-L (25 kDa) prevented us from clearly distinguishing these two bands (Fig. 2b). We could not observe the full-length Ran (51 kDa) under non-reducing conditions owing to the low expression level. The migration of the translation products was recorded by western blot under both reducing and non-reducing conditions using an anti-6x-His-tag monoclonal antibody and an anti-FLAG-tag monoclonal antibody, which recognized Ran-H and Ran-L, respectively. As expected, under reducing conditions, two protein bands were detected, corresponding to the molecular weights of Ran-H and Ran-L, respectively (Fig. 2e, Additional file 1: Fig. S1c). This result indicated that “self-cleavage” of the 2A peptide occurred during protein translation. Western blot analysis under non-reducing condition revealed a band of approximately 51 kDa, which is the expected size of a full-length Ran composed of both heavy chain and light chain. However, one additional protein band was detected at a position correlating to the size of heavy chain and light chain, respectively, which could be attributed to incomplete assembly of the heavy and light chains.

To avoid potential interference with the target proteins, we did not supplement the SD-2xSCAA medium with bovine serum albumin (BSA) although a previous study demonstrated that the addition of BSA in the fermentation medium could significantly reduce heterologous protein degradation by extracellular proteases in *S. cerevisiae* [41]. After confirming the expression and secretion of antibody fragments, we tested the influence of BSA on target protein production through culturing the strain LA.Nan in SD-2xSCAA medium and SD-2xSCAA medium without BSA. In accordance with the previous report, the strain growing in SD-2xSCAA medium resulted in a higher Nan production at different timepoints (Additional file 1: Fig. S3). This result indicated that the presence of BSA could to some extent protect the protein product from being degraded. Therefore, we added BSA to the medium throughout the following experiments to avoid potential protein degradation.

Taken together, all three strains produced protein bands of the expected molecular sizes, confirming that all selected antibody fragments could be expressed and secreted as soluble proteins in *S. cerevisiae*.

### **In vitro antibody activity assay**

**Fig. 3:**
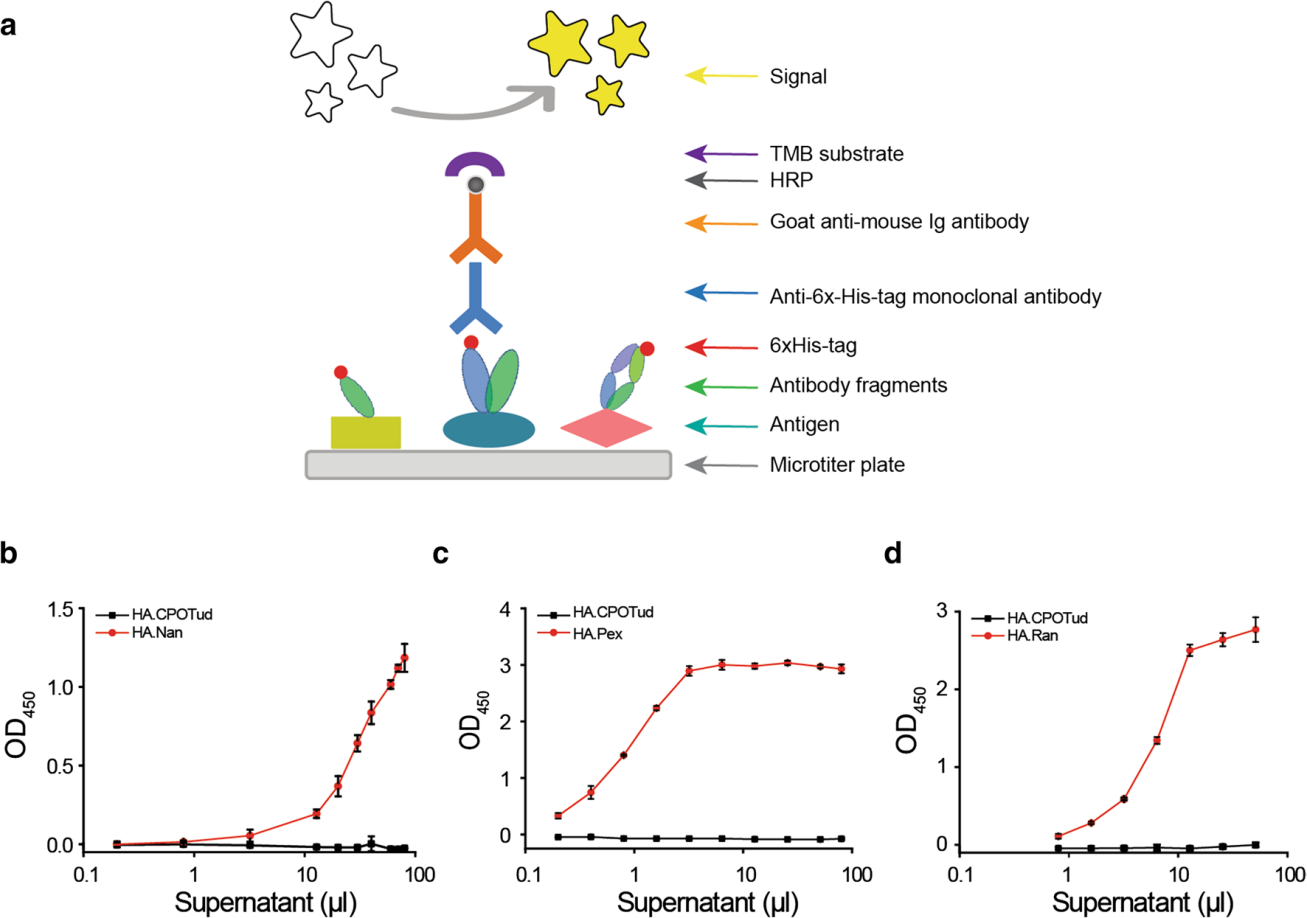
The binding activity of secreted antibody fragments in *S. cerevisiae*. **a** Schematic illustration of the sandwich ELISA format used here. **b** The binding activity of protein Nan to its corresponding antigen lysozyme from chicken egg white. **c** The binding activity of protein Pex to its corresponding antigen complement C5 from human serum. **d** The binding activity of protein Ran to its corresponding antigen human recombinant VEGF protein. Supernatants from HA.Nan, HA.Pex, HA.Ran and control HA.CPOTud strains were collected after 72 h of cultivation in SD-2xSCAA medium. Three antibody fragments were detected by ELISA signals (absorbance values are displayed as OD_450_) using anti-6x-His-tag monoclonal antibody, HA.CPOTud as a negative control. Results are average values ± SD of biological triplicates (Nan) and duplicates (Pex and Ran)

After the confirmation of protein expression and secretion of the selected antibody fragments, we proceeded to measure their biological activities. For this, an ELISA binding assay protocol was implemented to measure the protein-protein interaction between antibody fragments and their respective immobilized antigens. Figure 3a schematically illustrates the principle.

Since ELISA-based assays are highly specific and sensitive, we routinely purified the proteins from the culture supernatant using cobalt-based immunoprecipitation in order to avoid any interference by protein impurities in the medium. Subsequently, the purified proteins were serially diluted and tested by ELISA (Additional file 1: Fig. S4). The results showed an increase of absorbance values (OD_450_) in line with the increased concentration of proteins (Nan, Pex and Ran), while the control HA.CPOTud strain did not result in any absorbance at this wavelength, which indicated that the three heterologous proteins could specifically bind to their antigens.

In previous reports, the harvested culture supernatant was directly subjected to ELISA analysis [40, 42–44]. To evaluate if this was feasible here, we performed the respective experiment and proved that no biological activity was observed in the supernatant from control strain HA.CPOTud, which harbored the empty plasmid, indicating that impurities in the cultivation medium did not result in an unspecific reaction of the ELISA assay. Therefore, to simplify the analysis process and minimize protein loss, we omitted the purification process and instead directly collected the supernatant to measure binding activity. Consistent with above ELISA experiments using purified proteins (Additional file 1: Fig. S4), expression of three antibody fragments resulted in a marked increase of absorbance values (OD_450_) revealing the binding activity with increasing concentration of supernatant (Fig. 3b–d).

To obtain additional evidence about its biological specificity, we selected antibody fragment Nan (with C-6xHis-tag) as an example and further demonstrated its binding to the corresponding target protein lysozyme from chicken egg white (Lyz, with C-FLAG-tag) using a pull-down assay. Additional file 1: Fig. S5a illustrates the reaction process. Western blot analysis using anti-FLAG-tag monoclonal antibody resulted in detection of a Lyz band at the expected size (15 kDa) in the supernatant, which confirmed that the protein Lyz was secreted (Additional file 1: Fig. S5b). The interaction was demonstrated by the appearance of two protein bands in a reducing SDS-PAGE gel at molecular weights of approximately 17 kDa and 15 kDa (Additional file 1: Fig. S5c), corresponding to Nan and Lyz, respectively. The binding specificity was further confirmed by western blot using anti-FLAG-tag monoclonal antibody (Additional file 1: Fig. S5c). These data further supported the ELISA results.

Thus, these results demonstrated that all three antibody fragments, which were secreted from *S. cerevisiae*, showed biological activity in binding to their corresponding antigens.

### Comparison of secretion capacity in different mutant strains

We initially expressed and tested all antibody fragments in the HA strain, which was derived from B184 exhibiting high α-amylase secretion. To explore whether the improved secretion capacity of HA was generally applicable to produce different pharmaceutical proteins, we compared the production of the selected antibody fragments in different host strains, LA (the low-secretion strain), MA (the medium-secretion strain) and HA (the high-secretion strain), respectively.

A phenotypic change of the host strain can be determined by the properties of expressed proteins, and the growth rate presents an important characteristic of the physiological state. We therefore analyzed the cellular growth rate in a growth profiler prior to evaluating the secretory capacity. While the control strain LA containing an empty plasmid showed a typical diauxic growth behavior with a fermentative growth phase on glucose followed by a respiratory growth phase on ethanol, the protein expressing strains grew slower and did not display an obvious diauxic growth (Additional file 1: Fig. S6a). Moreover, all recombinant strains exhibited a longer lag phase and a decrease of the maximal specific growth rates compared to the corresponding strains containing the empty plasmid (Additional file 1: Fig. S6b). The growth rate depended both on the expressed antibody fragment and the strain background. Strain HA always showed the highest growth rate, followed by MA and then the parental strain LA (Additional file 1: Fig. S6b). The results indicate that the increase of protein secretory capacity does not exert a negative effect on cell growth. On the contrary, the mutant strains grew faster, consistent with the previous study [29].

**Fig. 4:**
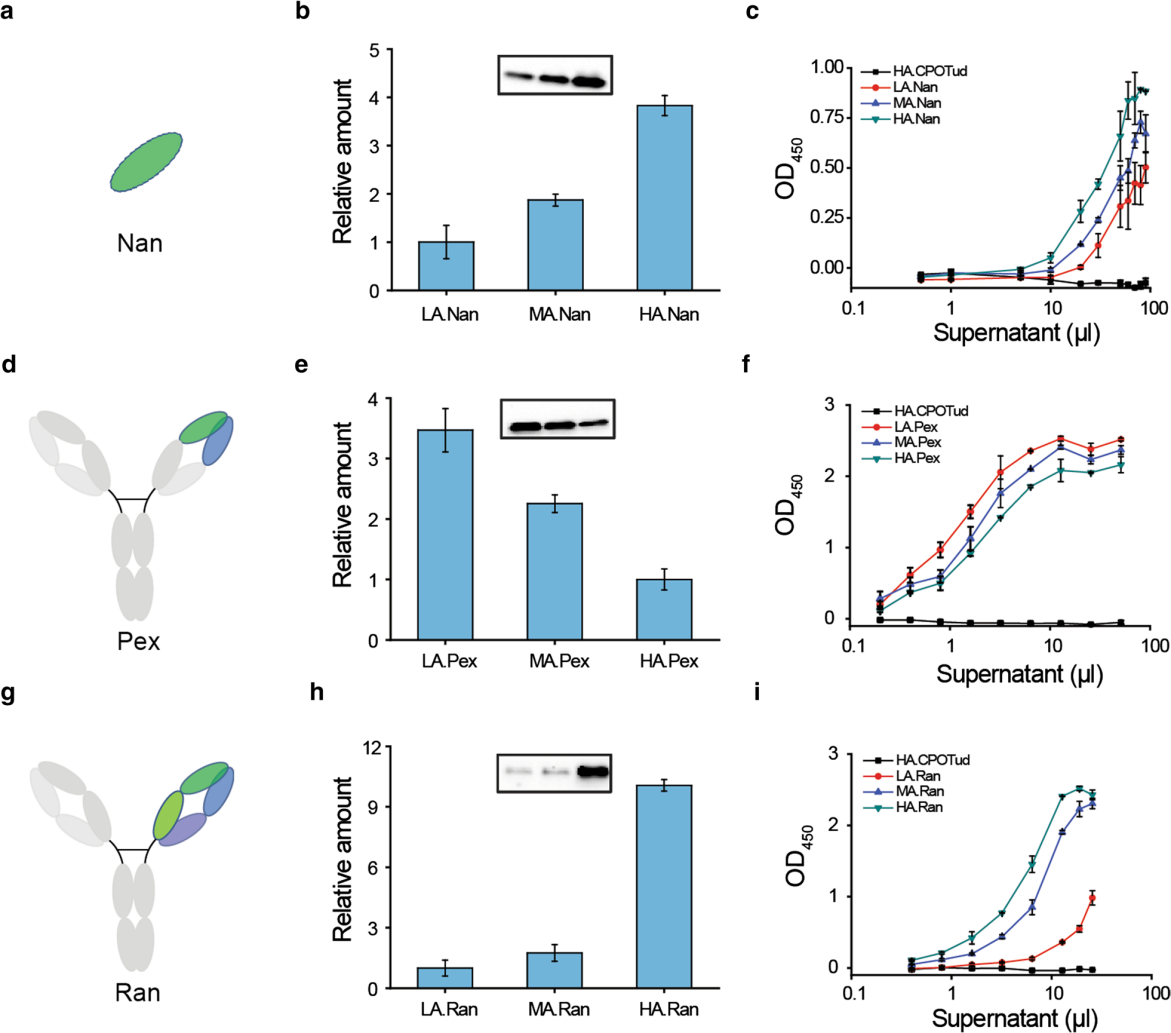
Comparison of protein secretion capacity in different mutant strains. Schematic illustration of antibody fragments (Nan (**a**), Pex (**d**) and Ran (**g**)). Supernatants from cultures of the antibody-expressing strains were collected after 72 h of cultivation and then processed. Western blot using anti-6x-His-tag antibody was performed under reducing (Nan (**b**) and Pex (**e**)) or nonreducing (Ran (**h**)) conditions. Data are expressed as average values ± SD of biological triplicates. The original images of the western blots are shown in Additional file 1: Fig. S7. For each protein, the secretion capacity in three yeast strains was evaluated (Nan (**c**), Pex (**f**) and Ran (**i**)). ELISA signals (absorbance values are displayed as OD_450_) for different recombinants were obtained with anti-6x-His-tag monoclonal antibody. The HA strain carrying empty plasmid CPOTud was used as the negative control. Results are shown as average values ± SD of biological triplicates (Nan, CPOTud) and duplicates (Pex and Ran)

Furthermore, we compared the protein secretion level in three selected background strains. Western blot analysis revealed that the amount of secreted Nan showed a significant increase in line with the evolution series. MA.Nan showed a 1.9-fold higher Nan titer, and HA.Nan could produce a 3.8-fold higher Nan titer compared to LA.Nan (Fig. 4a and b, Additional file 1: Fig. S7a). The amount of protein Ran presented a similar increasing tendency as Nan. MA.Ran, had a 1.8-fold higher production of Ran, and HA.Ran could produce 10.1-fold more Ran than LA.Ran (Fig. 4 g and h, Additional file 1: Fig. S7c). On the other hand, protein Pex showed an opposite trend. LA.Pex secreted a 3.5-fold higher amount compared with HA.Pex, while MA.Pex showed a 2.3-fold higher titer than HA.Pex (Fig. 4d and e, Additional file 1: Fig. S7b). The results were further confirmed by ELISA (Fig. 4c, f and i). These data demonstrated that the expression and secretion of Nan and Ran were positively correlated with the strains’ secretory capacity (as determined by α-amylase) [28], while the protein Pex exhibited the highest secretion level in the parental strain LA.Pex. It is noteworthy that the abundance of the heavy chain of Ran under non-reducing condition and the small band of Pex were consistent with the secretory level of Ran and Pex, respectively (Additional file 1: Fig. S7b and c). The results imply that the previously evolved secretory strains are suitable candidates for production of pharmaceuticals with dedicated selection and optimization, but this strategy may not be valid for all protein drugs.

### Transcriptional response of the different protein expression strains

To explore the fundamental changes triggered by the expression of the different pharmaceutical proteins, RNA-seq analysis was performed. Here, we chose two proteins (Nan and Pex) in two host backgrounds (LA and HA) for comparison due to their opposite secretory trend. The production of Nan was higher in the HA.Nan strain in comparison to the LA.Nan strain. On the contrary, the production of Pex was lower in the HA.Pex strain than in the LA.Pex strain (Fig. 4, Additional file 1: Fig. S7).

**Fig. 5:**
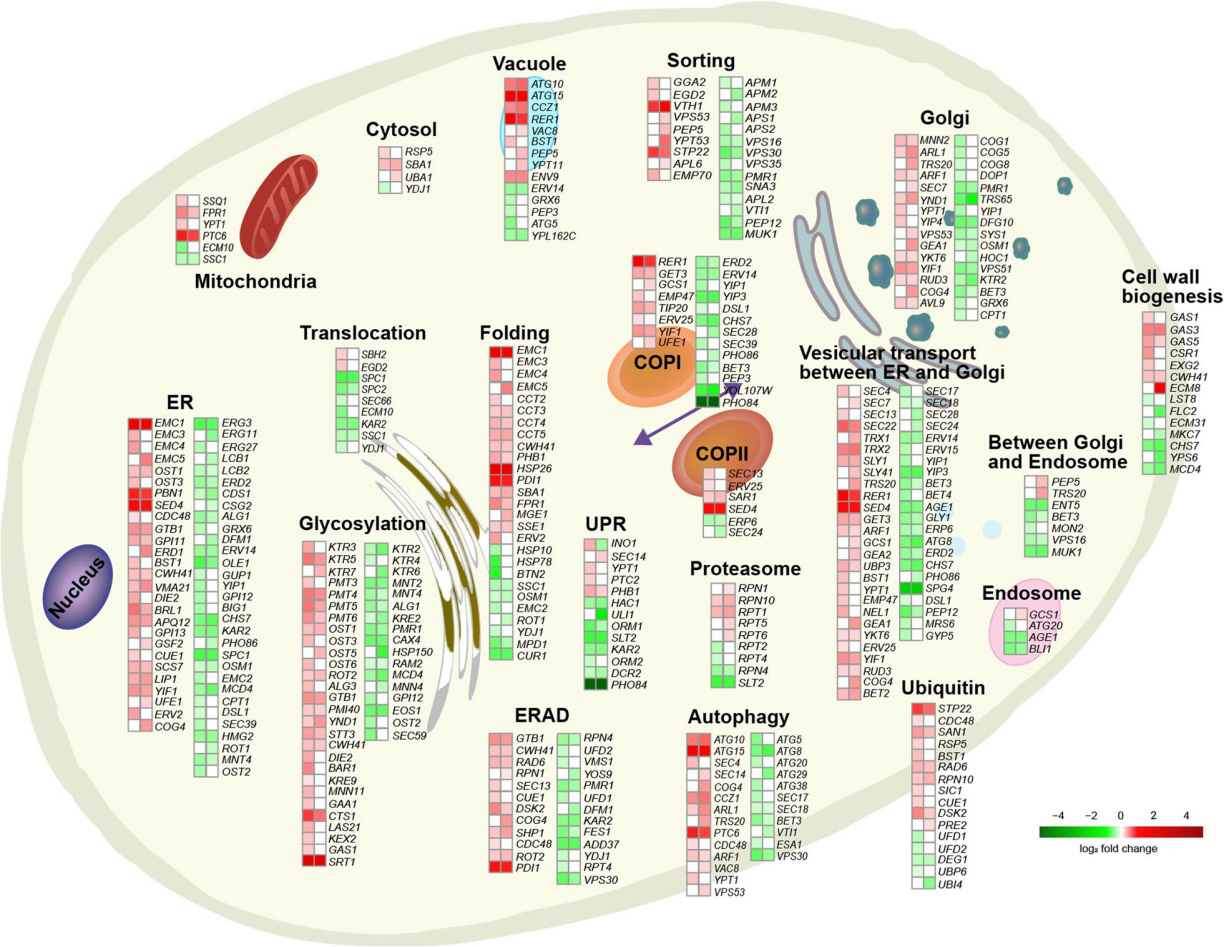
Transcriptional changes of genes related to the protein secretory pathway. Transcriptome data are shown as fold changes in Nan and Pex expressing HA and LA strains. The left column represents the comparison between HA.Nan and LA.Nan, while the right is the comparison between HA.Pex and LA.Pex. Red and green boxes represent the corresponding value of the log_2_ fold change (*p*-adj < 0.05) and white boxes represent *p*-adj ≥ 0.05. The same gene was classified into different subsystems based on the functional annotations

For analysis of the differential gene expression, we performed pairwise comparisons between the mutant and parental strains for Nan and Pex production: Nan-expressing strains (HA.Nan and LA.Nan) and Pex-expressing strains (HA.Pex and LA.Pex). The global expression pattern was characterized using principal component analysis (PCA). PCA showed a strong grouping of biological replicates, indicating a high degree of reproducibility (Additional file 1: Fig. S8a). The HA and LA strains showed distinct gene expression profiles. The first component of the PCA accounted for 75% of the variance and showed significant differences between the different background strains. PC1 clearly separated HA strain and LA strain independent of the produced proteins. For the LA strain, PC2 separated Pex and Nan, which was not the case for the HA strain. Differential gene expression analysis (*p*-adj and log_2_ fold change) identified that in HA.Nan 2289 genes, about 34% of all genes of *S. cerevisiae*, were significantly (*p*-adj < 0.05) upregulated or downregulated compared with LA.Nan (Additional file 1: Fig. S8b) and 1960 genes in HA.Pex vs. LA.Pex (Additional file 1: Fig. S8c). We also performed gene expression analysis of LA.Nan vs. LA.Pex and HA.Nan vs. HA.Pex, but only few significantly changed genes were discovered. Therefore, to identify common expression changes of HA.Nan vs. LA.Nan and HA.Pex vs. LA.Pex, significantly differentially expressed genes (*p*-adj < 0.05, log_2_ fold change < − 1 or > 1) were plotted in a Venn diagram (Additional file 1: Fig. S8d), and a total of 208 genes appeared in both HA.Nan vs. LA.Nan and HA.Pex vs. LA.Pex comparison (Additional file 2). To further investigate if the expression of the different proteins affected the protein secretory pathway, the expression level of genes in every subsystem was evaluated (Fig. 5). No notable gene expression change was identified when comparing the respective strains expressing the two proteins, suggesting that the genes in the protein secretory pathway were not influenced by the nature of the expressed protein. To further identify which genes might have important effects on antibody productivity, we first selected 2670 significantly changed genes in strain HA.Nan vs. LA.Nan (group I, *p*-adj < 0.05) or strain HA.Pex vs. LA.Pex (group II, *p*-adj < 0.05). Further, to identify differentially changed genes between group I and II, we selected genes based on the formula abs (log_2_ fold change I–log_2_ fold change II) > 1 and obtained 100 genes. Of these, we selected ten directionally significantly changed genes for either deletion or overexpression according to the directional change in strain HA.Nan vs. LA.Nan (Additional file 1: Fig. S9a). And the genetic modifications were performed in strain LA.Pex. These ten genes were *YDR344C*, *STL1*, *HXT3*, *TKL2*, *FRE4*, *KNS1*, *GIT1*, *AZR1*, *VMA1*, and *MIN7*. Among them, manipulations of 5 genes, i.e. deletion of *STL1*, deletion of *TKL2*, overexpression of *GIT1*, overexpression of *AZR1, and* overexpression of *KNS1*, showed a potential positive effect using an ELISA assay (Additional file 1: Fig. S9b). However, the standard deviations of some of the results were rather high. We then performed western blot to further confirm their effects. Only overexpression of *KNS1* showed a distinct positive effect on Pex expression in strain LA (Additional file 1: Fig. S9c and S9d). *KNS1* encodes a protein kinase involved in TOR signaling and regulation of RNA polymerase III [45].

**Fig. 6:**
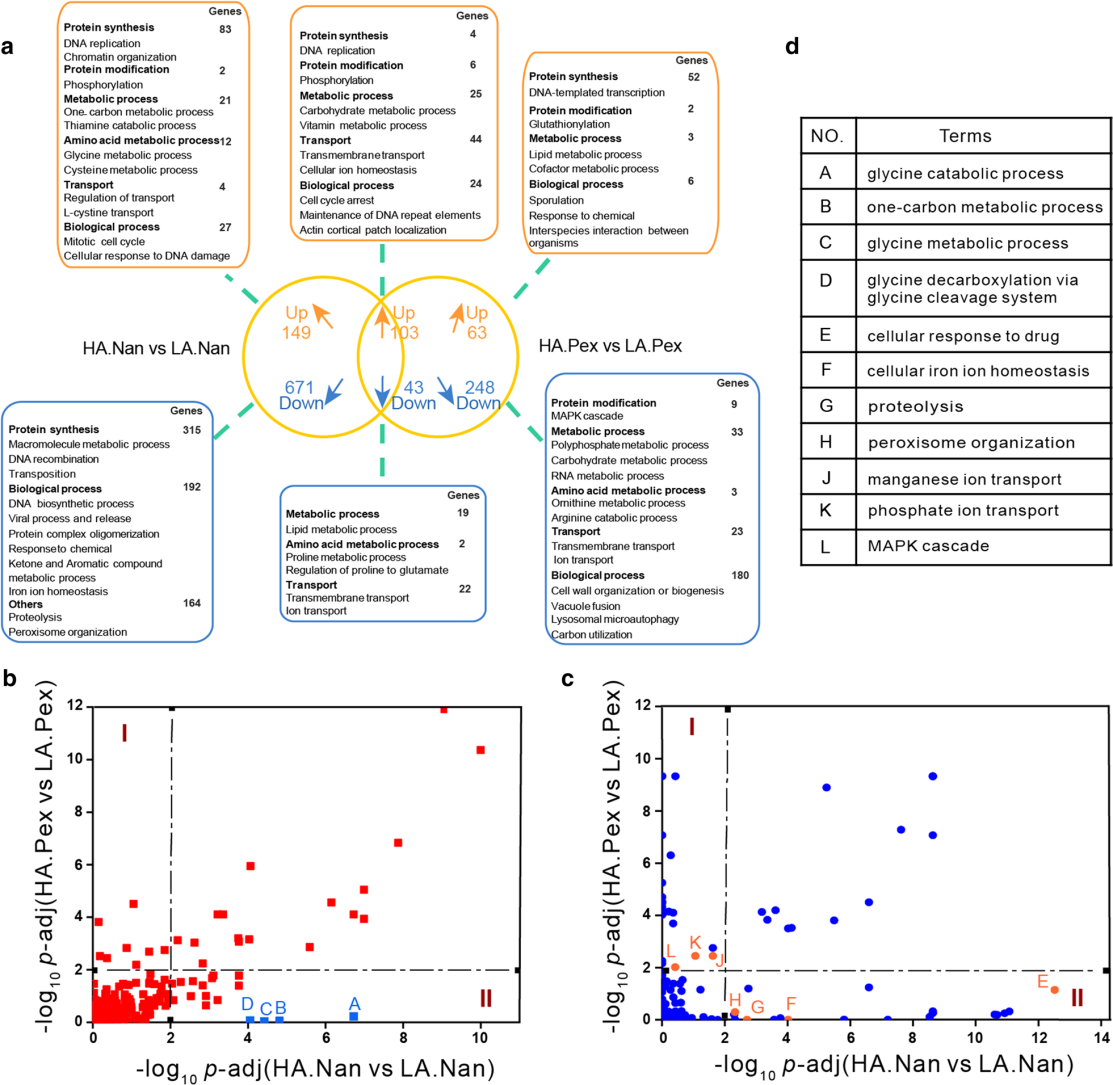
GO term analysis for Nan and Pex expression in the mutant and parental strains. **a** Cellular responsive bioprocesses affected by different protein expression. Up and down represent up-regulation and down-regulation. Numbers represent the amount of genes in the up- and down-regulated terms. We compared the same terms between HA.Nan vs. LA.Nan and HA.Pex vs. LA.Pex based on up-regulation (**b**) or down-regulation (**c**). We focused on terms in part I and terms in part II that show significant differences for the expression of only one protein. The squares and dots on the plot represent the same terms. Dashed lines correspond to *p*-adj = 0.01. 0–2 indicates no significant differences (*p*-adj > 0.01) and > 2 indicates a significant difference (*p*-adj < 0.01). The terms A-L are marked on the plot and listed in the table (**d**)

Gene Ontology (GO) term analysis is commonly used to identify enriched cellular responsive bioprocesses affected by different perturbations. First, we calculated the commonly changed bioprocesses representing consistent cellular responses during Nan and Pex expression (Fig. 6a). The up-regulated terms were related to protein synthesis and modification, cellular transport and certain metabolic processes, while the down-regulated terms were related to lipid and amino acid metabolic processes, and transport. As the HA and LA background strains exhibited different production capacities for the two proteins, with the aim to gain more insight in differently influenced biological processes caused by these two proteins, we analyzed the inconsistent responses. From this analysis, we found that the significantly (*p*-adj < 0.01) up-regulated terms were concentrated on glycine and cysteine metabolism, one-carbon and thiamine metabolism, and cell cycle in the HA.Nan strain compared to the LA.Nan strain, while the significantly down-regulated terms were focused on protein synthesis (Fig. 6a, Additional file 3). In strain HA.Pex vs. LA.Pex, the terms related to sporulation, and stress-associated responses were significantly upregulated, while for down-regulated terms, MAPK cascade, ornithine and arginine metabolism, and carbohydrate and polyphosphate metabolic processes were enriched (Fig. 6a, Additional file 3).

To identify which biological processes exhibited significant differences, we compared the same terms between HA.Nan vs. LA.Nan and HA.Pex vs. LA.Pex based on up- or down-regulation. To explore the key differences, we focused on the terms which showed significant changes (*p*-adj < 0.01) upon expression of one protein but not upon expression of the other protein (*p*-adj ≥ 0.01) (Fig. 6b and c). Concerning up-regulated terms, we found that the most significant differences for both proteins in different background strains were mainly related to the glycine catabolic process, one-carbon metabolic process, glycine metabolic process and glycine decarboxylation via glycine cleavage system. The four processes were significantly upregulated (*p*-adj < 0.01) in the HA.Nan vs. LA.Nan while they were not significantly changed in HA.Pex vs. LA.Pex (Fig. 6b). Since both proteins contain a similar fraction of glycine residues, a higher glycine requirement for protein production would therefore not provide an explanation for this observation. To validate the potential effect of glycine metabolism on cellular production of antibody fragments Nan and Pex, we changed the concentration of glycine in the SD-2xSCAA fermentation medium. However, neither increase nor decrease of the glycine concentration in the medium revealed a clear trend in their effect on Nan and Pex production in strains LA and HA (Additional file 1: Fig. S10). Concerning the down-regulated terms, we found that the cellular response to drugs, cellular iron ion homeostasis, peroxisome organization, and proteolysis were significantly downregulated (*p*-adj < 0.01) in strain HA vs. LA during the expression of Nan, and ion transport and MAPK cascade were significantly downregulated (*p*-adj < 0.01) during the expression of Pex (Fig. 6c). These significantly downregulated terms in strain HA vs. LA were related to the stress response, while they were not significantly downregulated (*p*-adj < 0.01) in strain HA.Pex in comparison to LA.Pex. This indicated the expression of Pex might cause an increased cell stress in comparison with Nan.

Gene set enrichment analysis also revealed that, for the HA.Nan strain, pathways involved in amino acid metabolism were significantly downregulated (*p*-adj < 0.05) in comparison to LA. Nan and therefore could result in a decreased protein synthesis (Additional file 1: Fig. S11a and b). This would relieve ER stress by reducing the load of newly synthesized proteins.

## Discussion

During recent decades, the production of antibody fragments has drawn increasing interest because they not only retain antigen binding properties but are also easier and less costly produced in microbial systems. Due to their small size, they can penetrate tissues and tumors more rapidly and more efficiently than the full size mAbs, which are used in tumor imaging, radiotherapy of cancers, and cardiovascular applications [6]. In our study, three antibody fragments with different configurations (such as disulfide bonds and molecular weights, Table 1) were successfully expressed in the high-level secretory platform strain HA.

In the process of antibody expression, the ratio of heavy and light chains is crucial for the final antibody production and quality. This is also true for the expression of Fab fragments. Thus, an emerging challenge is to precisely control the relative expression level of both chains. 2A peptides, derived from viruses, have around 20 amino acids. The ribosomal skip occurs co-translationally between the last two amino acids, which results in equal amounts of the co-expressed proteins [46, 47]. Owing to highly efficient cleavage, 2A peptides have been used in various eukaryotic systems, including mammalian cell lines, plants and yeasts, and also successfully applied to control the performance of multi-step biosynthetic pathways [48, 49]. A study using human embryonic kidney 293 cells showed that a full-length and functional monoclonal antibody was successfully expressed with a 2A-mediated expression system [42]. The cleavage efficiency of a 2A peptide may be affected by the nature of the protein expressed. Of the 2A peptides identified to date, four with varied cleavage efficiency have been commonly used in biomedical research: F2A from foot-and-mouth disease virus, E2A from equine rhinitis A virus, P2A from porcine teschovirus-1 and T2A from Thosea asigna virus [50]. In this work, we used the 2A peptide from Thosea asigna virus. In addition to a band representing the assembled antibody fragment, we observed the single heavy chain and light chain bands under non-reducing conditions, which might have resulted from incomplete assembly of heavy chain and light chain. The choice of the different signaling peptides for Ran-H and Ran-L may have resulted in differences in secretion efficiency of the two proteins although a previous study indicates that it should be similar for these two signaling sequences [27]. In addition to the ratio of the two chains, the folding and assembly processes of mAbs and antibody fragments also depend on multiple disulfide bonds. Previous studies indicated that the formation of disulfide bonds between light chain and heavy chain is one of the main limitations of Fab quality and production [51, 52]. In *Pichia pastoris*, owing to the inefficient formation of intermolecular disulfide bond, only about 30% of Fab fragments were produced [44]. The requirement for formation of several disulfide bonds (Table 1) specifically the disulfide bond between C_H_1 and C_L_ in Ran probably limited its efficient assembly and resulted in low production (Fig. 2c–e, Additional file 1: Fig. S1). An oxidizing environment is beneficial to the formation of disulfide bonds [53, 54]. For providing the oxidizing environment and therefore optimizing functional Ran production, it would be possible to examine the influence of culture conditions (i.e. dissolved oxygen, pH value or supplying oxidizing substances in the medium) or diminish the reductive pathways that accompany the secretion process. Such strategies might however also affect cell growth and/or viability. In addition, achieving successful mAb assembly requires adopting more engineering strategies, e.g. improving the assembling rate of the heavy chain and light chain in the ER and ensuring equal translocation effectiveness.

In our study, although three antibody fragments could be functionally expressed and secreted in yeast HA, an effective secretion strategy for one protein might not be generally applicable for improving production of another protein due to various factors that could affect the capacity of the yeast secretory machinery [27]. For example, the properties of the target protein, selection of genetic expression systems, capacity and efficiency of transcription and translation, protein folding and post-translational modifications, and vesicle trafficking, may potentially represent bottlenecks that limit effective secretion of heterologous proteins [55]. We therefore evaluated the secretion capacity of these three antibody fragments in three different engineered strains. These strains have been assessed for the production of different enzymes but not for antibody fragments. Our results showed that the amount of secreted proteins differentially correlated with the secretory capacity of the selected host strains. The secretion of Pex decreased with the increase of the secretory capacity, while the amounts of other two proteins were consistent with the increased secretory capacity [28]. The protein secretory pathway in eukaryal cells is a complex system and any change in the subsystems (i.e. translation and folding behavior) could lead to different levels of cell stress, cause different perturbations to protein secretion and hence result in different production levels. In this work, GO-term analysis was performed to examine different perturbations during the expression of Nan and Pex in the low and high secretory host strains LA and HA. We found a significant difference for protein synthesis, protein modification, and amino acids metabolic process (Fig. 6a) which were probably related to protein secretion capacity. GO-term analysis also revealed that during the expression of Pex, the GO-terms related to stress responses were not significantly downregulated in HA.Pex vs. LA.Pex, while HA.Nan vs. LA.Nan exhibited the opposite, coinciding with the slight reduction of specific growth rate in the Pex expressing strain compared to the Nan and Ran expressing strains (Additional file 1: Fig. S6b). It is interesting that we did not see these transcriptional changes in the original alpha-amylase strains, indicating an effect specifically caused by antibody fragment production [29, 56]. To identify the effects of differentially changed genes on antibody fragment production, we tested ten significantly changed genes and found that the overexpression of *KNS1* had a positive effect on Pex expression in strain LA (Additional file 1: Fig. S9). As an important downstream element of TOR-dependent signaling, the kinase Kns1 is differentially expressed and hyperphosphorylated to regulate ribosome and tRNA synthesis in response to cellular stress or nutrient limitation [45], which further indicates an important role of the stress response in antibody fragment production. An additional phenomenon should be taken into account; only very small colonies appeared on the plates when the plasmid that encoded Pex was transferred into strains LA and MA. We suspect that the expression of *Pex* could compete for cellular resources and thus alter the intracellular metabolism, which might cause a compromised cell growth. In addition, from GO-term analysis, we found that glycine metabolism related terms were significantly upregulated in the strain HA.Nan vs. LA. Nan, but not in strain HA.Pex vs. LA.Pex. Upon changing the concentration of glycine in the fermentation medium, despite not finding a clear trend in the effect on the production of the two antibody fragments in strains LA and HA, we observed a varied production level of antibody fragments (Additional file 1: Fig. S10). All these data indicated that efficient protein production also requires optimization of cellular metabolism to ensure sufficient supply of precursors and energy for target protein production and allow the cells to quickly respond to different cellular perturbations. We therefore conclude that both the stress and the allocation of cellular resources caused by Pex expression are presumably responsible for the low efficiency in secretion of Pex in the HA strain.

In summary, we demonstrated the secretion and biological specificity of three antibody fragments with different configurations (Table 1), evaluated the secretion capacity for these proteins in different mutant strains and explored the potential reasons for the differences in production capacity by transcriptomics analysis. The results indicated that factors limiting the protein production not only reside in the secretory pathway but might also be related to the allocation of cellular resources. Future studies may focus on evaluating amino acid metabolism and activating stress responses to unfavorable environmental changes for improving recombinant protein production. It is important to move forward by exploring and rewiring these processes, as a foundation for future large-scale production of pharmaceutical proteins.

## Methods

### Media and culture conditions

*Escherichia coli*  was cultivated in LB medium supplemented with final concentrations of 100 mg/L ampicillin or 50 mg/L kanamycin at 37 °C. Yeast strains were normally cultivated in YPD medium containing 10 g/L yeast extract (Merck Millipore), 20 g/L peptone (Difco), and 20 g/L glucose (VWR). Strains without plasmids were cultivated in YPE medium containing 10 g/L yeast extract, 20 g/L peptone, 10 g/L ethanol, and 0.5 g/L glucose. Strains without the α-amylase plasmid were verified on starch agar plates, which contained 0.04 g/L glucose, 10 g/L starch, 6.9 g/L yeast nitrogen base without amino acids (Formedium), and 20 g/L agar (Merck Millipore). The starch and ethanol agar plate consisted of 0.04 g/L glucose, 10 g/L starch, 6.9 g/L yeast nitrogen base without amino acids, 10 g/L ethanol, 790 mg/L complete supplement mixture (CSM) (Formedium), and 20 g/L agar. Strains under amdSYM-selective conditions were cultivated in SM-Ac medium containing 3 g/L KH_2_PO_4_, 0.5 g/L MgSO_4_·7H_2_O, 0.6 g/L acetamide, 6.6 g/L K_2_SO_4_, 1 ml/L of a vitamin solution, 1 ml/L of a trace element solution, and 20 g/L glucose [57]. The plates were prepared by adding 20 g/L agar to the medium. All yeast strains were cultured at 30 °C throughout this study.

Tube cultivations or shake flask batch fermentations for protein production were carried out in SD-2xSCAA medium containing 20 g/L glucose, 6.9 g/L yeast nitrogen base without amino acids, 190 mg/L Arg, 400 mg/L Asp, 1,260 mg/L Gln, 130 mg/L Gly, 140 mg/L His, 290 mg/L Ile, 400 mg/L Leu, 440 mg/L Lys, 108 mg/L Met, 200 mg/L Phe, 220 mg/L Thr, 40 mg/L Trp, 52 mg/L Tyr, 380 mg/L Val, 1 g/L BSA, 5.4 g/L Na_2_HPO_4_, and 8.56 g/L NaH_2_PO_4_·H_2_O (pH 6.0). Single colonies were used to inoculate 14-mL tubes carrying 1.5 mL of liquid medium, which were subsequently incubated with 200-rpm agitation for 24 h. Precultures were then used to inoculate 100-mL unbaffled shake flasks carrying 20 mL of medium at an initial optical density at 600 nm (OD_600_) of 0.05 and cultivated at 200 rpm for 72 h.

For recording cell growth, engineered strains were analysed with continuous orbital shaking using a growth profiler 960 (EnzyScreen). Precultures were used to inoculate a 96-well flat-bottom microplate (250 µl cultures) at an initial OD_600_ of 0.05 and OD_600_ values (referred to as OD_600_ equivalents) were measured with an interval of 30 min for 72 h.

### Strains and plasmids

*E. coli* DH5α was used to construct and propagate the recombinant plasmids. All the *S. cerevisiae* strains were derived from the parental strain CEN.PK 530.1 C by UV mutagenesis [28] and plasmids are listed in Additional file 4: Table S1. All the primers were ordered from Eurofins Genomics and are listed in Additional file 4: Table S2. All the codon optimized heterologous genes were synthesized from Genscript and are listed in Additional file 4: Table S3. DNA sequencing services were provided by Eurofins Genomics.

The plasmid CPOTud as the backbone was digested by restriction enzymes KpnI and NheI. Gene Ran encoded two chains, part of the heavy chain with a 6xHis-tag (Ran-H) and the light chain with a FLAG-tag (Ran-L) at their respective C-termini. To co-express the two chains in one transcript, an 18-amino acid 2A self-processing peptide from Thosea asigna virus (EGRGSLLTCGDVEENPGP) was added at the C terminus of Ran-H [39]. To eliminate possible adverse effects caused by the remaining 2A residues, the construct also contained an additional Kex2 cleavage site (KR), which was located between the heavy chain and the 2A sequence. A GSG linker for higher 2A cleavage efficiency by creating greater flexibility was inserted between the additional Kex2 cleavage site and 2A peptide [40]. Ran-H and Ran-L were added with an alpha-factor leader (three glycosylation sites) and synthetic leader Yap3-TA57 (no glycosylation sites) at their N terminus [58]. The other three single peptides, Nan, Pex and Lyz (with 6xHis-tag, 6xHis-tag and FLAG-tag, respectively), were synthesized with an alpha-factor leader. All these assembled fragments were digested by KpnI and NheI, and then integrated into the backbone plasmid under the control of a strong *TPI1* promoter, resulting in pCP-Nan, pCP-Pex, pCP-Ran, and pCP-Lyz plasmids (Fig. 2a).

For obtaining the antibody fragment producing strains, the α-amylase expression plasmids were first eliminated from the mutant strains by a series of selective transfers in YPE medium. In brief, one colony from each recombinant strain (AAC, MH34 and B184) was used to inoculate 1.5 mL of YPE medium. 10 µL of the culture were transferred into new YPE medium twice, and then streaked on a YPE plate. Loss of the α-amylase plasmid was confirmed by checking for the inability to use starch from starch agar plates indicated by a lack of growth and lack of a halo on the starch agar plate. Subsequently, the constructed plasmids pCP-Nan, pCP-Pex, pCP-Ran were used to transform the plasmid-depleted host strains LA, MA and HA, respectively.

For single gene deletions and promoter replacements, amdS was used as a marker for selection of transformants, which was amplified from plasmid pUG-amdSYM [55]. Gene deletion cassettes were assembled by the fusion of about 700 bp homologous sequences upstream to the target gene, amdS marker, and about 700 bp homologous sequences downstream to the target gene. Gene promoter replacement constructs were assembled by the fusion of about 700 bp homologous sequences upstream to the target gene, amdS marker, the strong *TEF1* promoter, and the target gene. The cassettes were transformed into the host strain LA.Pex and selected on the SM-Ac plates. The lithium acetate method was used for transformations [59].

### SDS-PAGE and western blot

For SDS-PAGE and western blotting analysis, strains were grown in SD-2 × SCAA medium without BSA if not specified. After cultivation, the supernatant was collected by centrifugation for 3000*g*, 4 min. For the visualization of the amount of secreted Pex, 1.8 mL of supernatant was concentrated 60-fold using the 10 K MW Pierce Concentrator PES (Thermo Scientific). For avoiding a too high amount of protein Nan, the supernatant was diluted 10-fold using phosphate-buffered saline (PBS) buffer (8 g/L NaCl, 0.2 g/L KCl, 1.44 g/L Na_2_HPO_4_, 0.24 g/L KH_2_PO_4_, pH 7.4). For reducing SDS-PAGE, 10 µL of cell supernatants were mixed with 9 µL loading dye and 1 µL 2-mercaptoethanol. For non-reducing SDS-PAGE, 10 µL of cell supernatants were mixed with 10 µL loading dye. In both cases, samples were heated at 98 °C for 5 min and then loaded on 4–20% tris-glycine pre-cast gels (Bio-Rad) and run for 1 h at 120 V in Tris/Glycine/SDS electrophoresis buffer (Bio-Rad). Then the samples were either visualized by staining with Coomassie Blue G-250 or further processed for western blot as described below.

Proteins in polyacrylamide gels were transferred to PVDF membranes using a Trans-Blot Turbo Transfer System (Bio-Rad). The membranes were washed with PBS and blocked for 1 h using 5% nonfat milk (w/v) in PBST (PBS containing 0.05% Tween-20). Membranes containing proteins were incubated with 1:2000-diluted anti-6x-His-tag monoclonal antibody (Thermo Scientific) or FLAG-tag monoclonal antibody (Thermo Scientific) in 1% milk (w/v)-PBST overnight at 4 °C and then with 1:1000-diluted polyclonal goat anti-mouse immunoglobulin antibody conjugated with horseradish peroxidase (HRP) (Dako) in 1% milk (w/v)-PBST for 1 h. Protein bands were visualized by exposure on a ChemiDoc XRS image analyzer (Bio-Rad) after the membranes had been treated with an enhanced chemiluminescent horseradish peroxidase substrate – SuperSignal West Dura Extended Duration Substrate (ThermoFisher Scientific). The intensity of the bands was analyzed using Image Lab 6.0.1.

### ELISA

An enzyme-linked immunosorbent assay (ELISA) was used to measure antigen-binding activity. MaxiSorp 96 well ELISA plates were coated with 100 µL/well of 5 µg/mL antigen in 0.05 M sodium carbonate buffer (1.515 g/L Na_2_CO_3_, 3 g/L NaHCO_3_, pH 9.6) overnight at 4 °C. All the antigens were obtained from Sigma-Aldrich, including lysozyme from chicken egg white, Complement C5 from human serum, as well as human recombinant VEGF protein. After removal of the coating solution, plates were washed twice with 200 µL PBST and blocked with blocking buffer (1% BSA in PBST) at room temperature for 1 h. Following four times of washing with 200 µL of PBST buffer, plates were incubated with protein samples for various processing conditions (The supernatant for protein Pex was 130 times concentrated, supernatant for protein Ran was not concentrated, and supernatant for protein Nan was 60 times diluted.) at successively varied volume (0 µL to 80 µL) at room temperature for 1 h. After washing four times with the same buffer to remove unbound protein, 100 µL of anti-6x-His-tag monoclonal antibody at various dilutions (1:500, 1:1000, 1:2000, 1:3000, 1:4000, 1:5000) in blocking buffer was added to each well. Plates were washed and incubated with 100 µL of various dilution of polyclonal goat anti-mouse immunoglobulins/HRP antibody (1:500, 1:1000, 1:2000, 1:3000) in blocking buffer at room temperature for 1 h. To avoid forming red precipitate in the ELISA plate wells, we identified the optimal reaction condition, which was 1:5000 dilution for the anti-6x-His-tag monoclonal antibody and 1:3000 dilution for the polyclonal goat anti-mouse immunoglobulins/HRP antibody. For detection, 100 µL of the 3, 3′, 5, 5′-tetramethylbenzidine (TMB) substrate was added and the activities of HRP were quantified by measuring the absorbance at 450 nm using a plate reader- FLUOstar® Omega (BMG labtech).

### Immunoprecipitation and pull-down assay

Biological activity of the antibody fragment Nan was also demonstrated by monitoring the aggregation and disaggregation in the presence or absence of its target. Three yeast strains, containing three plasmids, which encoded Nan, its target Lyz and empty plasmid, were cultured in SD-2 × SCAA for 72 h. The supernatants were harvested by centrifugation at 3000*g* for 4 min. For clearly visualizing on SDS-PAGE gel, the supernatant containing the antigen Lyz was concentrated eight times. The pull-down assay was carried out using Dynabeads™ magnetic bead-based technology (Invitrogen) according to the manufacturer’s protocol. Briefly, sample 1 was prepared in a volume of 700 µL 1 × Binding/Wash Buffer (2  ×  Binding/Wash Buffer: 100 mM sodium phosphate pH 8.0, 600 mM NaCl, 0.02% Tween™ -20), and incubated with cobalt-based Dynabeads in the 1.5 mL-microcentrifuge tubes on a rotary shaker (Stuart) at 4 °C for 10 min. Then the tubes were put on a magnet for 2 min. After discarding the supernatants, the beads were washed four times with 300 µL 1 × Wash Buffer by placing the tubes on a magnet for 2 min.

Similarly, sample 2 was prepared in a volume of 700 µL 1 × Pull-down Buffer (2 × Pull-down Buffer: 6.5 mM Sodium-Phosphate pH 7.4, 140 mM NaCl, 0.02% Tween™ -20), and then added to above protein A-treated beads. Following incubation at 4 °C for 30 min on the rotary shaker, the beads were washed four times. To elute the protein, the beads were suspended in 100 µL of Elution Buffer (300 mM imidazole, 50 mM sodium phosphate pH 8.0, 300 mM NaCl, 0.01% Tween™ -20) and incubated on a Sample Mixer (Eppendorf) for 10 min. The elution fractions were analyzed by SDS-PAGE and transferred onto a PVDF membrane for verifying the presence of the FLAG-tag.

### Transcriptome profiling

Cell samples for transcriptomic analysis were taken from biological triplicates at the early exponential phase (OD_600_ ≈ 1). 10 OD_600_ of cells were added to ice-chilled 15-mL falcon tubes containing approximately 10 mL ice and then immediately pelleted in a 4 °C pre-chilled centrifuge for 5 min at 2500 rpm. After centrifugation, supernatants were discarded, and cell pellets were snap frozen in liquid nitrogen and stored at −80 °C until further analysis. RNA was extracted and purified using a Qiagen RNeasy Mini Kit (Qiagen) with a FastPrep-24 homogenizer (MP Biomedicals) including DNA degradation according to manufacturer’s protocol. RNA integrity was assessed using a 2100 Bioanalyzer (Agilent Technologies). RNA concentration was determined by a Qubit RNA HS Assay Kit (Thermo Scientific) and cross-verified with a NanoDrop 2000 (Thermo Scientific). Libraries were prepared using an Illumina TruSeq Stranded mRNA Library Prep Kit (Illumina). Paired-end sequencing (2 × 150 bp) was carried out on a single lane on a HiSeq 2500, according to the user’s manual. The Novo Nordisk Foundation Centre for Biosustainability (Technical University of Denmark) performed the RNA sequencing and library preparation. Read pairs were quality controlled from 24.7 to 29.8 million and mapped to the *S. cerevisiae* reference genome (Ensembl R64-1-1) using STAR. RNA-seq data were processed using the nf-core RNAseq pipeline (SciLifeLab), available at https://github.com/nf-core/rnaseq. Analysis of differential expression was performed using the DESeq2 package in the R programming language. Reporter analysis on differential gene expression levels (log_2_ fold change) and corresponding significance levels (*p*-adj, calculated by the Benjamini–Hochberg method) were used as input. Reporter analysis on GO terms was carried out by using the Platform for Integrative Analysis of Omics (PIANO) R package with GO terms information from the SGD database (http://www.yeastgenome.org). The KEGG pathway functional categories enrichment analysis was performed by using online software David (Database for Annotation, Visualization, and Integrated Discovery), available at https://david.ncifcrf.gov/.

## Supplementary Information

**Additional file 1: Figure S1.** The original images of the western blots of supernatant from recombinant HA host strains. **Figure S2.** Expression of Pex after R130K codon mutation. **Figure S3.** Influence of BSA in the medium on protein production. **Figure S4.** Binding activity of Nan, Pex and Ran fragments after immunoprecipitation. **Figure S5.** Pull-down assay for assessing the biological activity. **Figure S6.** Growth phenotype of different strains expressing antibody fragments. **Figure S7.** The original images of the western blots of three different host strains for each protein. **Figure S8.** Global transcriptional response to the expression of Nan and Pex in LA and MA strains. **Figure S9.** The impact of modified genes on Pex secretion in the LA.Pex strain. **Figure S10.** Effect of changes in glycine concentration on protein production. **Figure S11.** Gene set enrichment analysis.

**Additional file 2.** Common significantly differentially expressed genes in both HA.Nan vs LA.Nan and HA.Pex vs LA.Pex.

**Additional file 3.** Genes with differential biological responses caused by the expression of Nan and Pex.

**Additional file 4: Table S1.** List of strains and plasmids. **Table S2.** List of oligonucleotide primer sequences. **Table S3.** Codon-optimized heterologous genes.

## Data Availability

The RNA-seq raw data can be downloaded from the Gene Expression Omnibus (GEO) with the access number GSE179391. The datasets used and analyzed during the current study are available from the corresponding author on reasonable request.
