# Supplementary material for: Expression of antibody fragments in Saccharomyces cerevisiae strains evolved for enhanced protein secretion
Source: Microb Cell Fact. 2021 Jul 14;20:134. doi: 10.1186/s12934-021-01624-0 (PMC8278646; doi:10.1186/s12934-021-01624-0)
Supplement: Supplementary file 1 — Additional file 1: Figure S1. The original images of the western blots of supernatant from recombinant HA host strains. Figure S2. Expression of Pex after R130K codon mutation. Figure S3. Influence of BSA in the medium on protein production. Figure S4. Binding activity of Nan, Pex and Ran fragments after immunoprecipitation. Figure S5. Pull-down assay for assessing the biological activity. Figure S6. Growth phenotype of different strains expressing antibody fragments. Figure S7. The original images of the western blots of three different host strains for each protein. Figure S8. Global transcriptional response to the expression of Nan and Pex in LA and MA strains. Figure S9. The impact of modified genes on Pex secretion in the LA.Pex strain. Figure S10. Effect of changes in glycine concentration on protein production. Figure S11. Gene set enrichment analysis. [file 12934_2021_1624_MOESM1_ESM.docx]

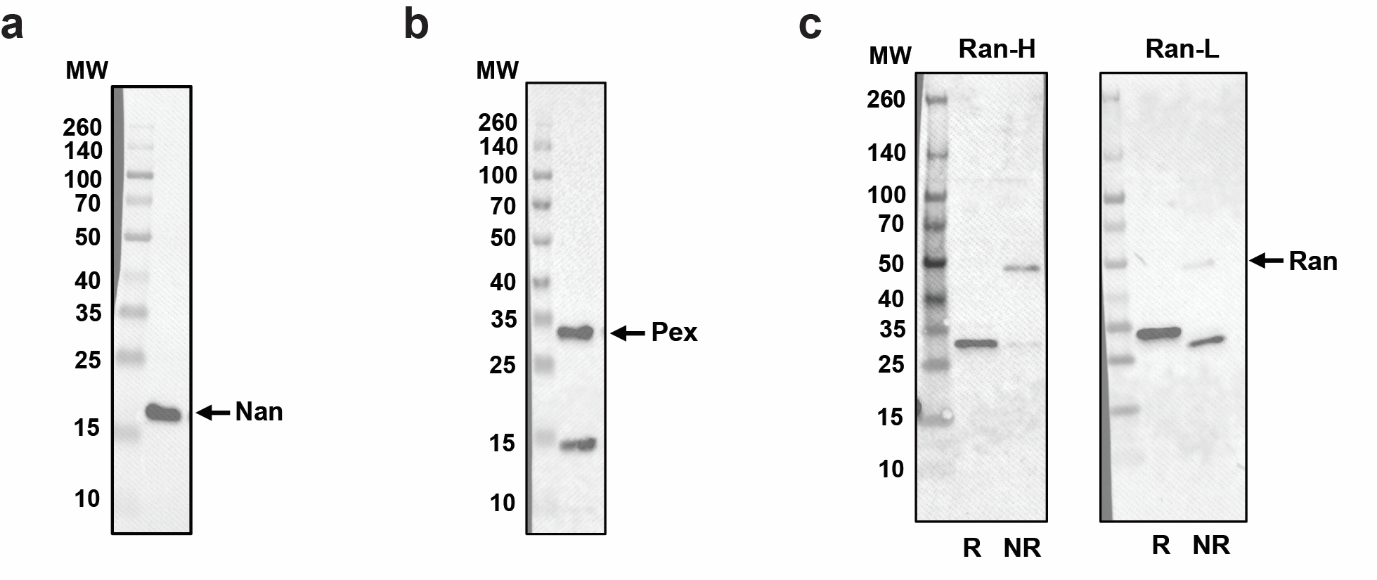


**Figure S1** The original images of the western blots of supernatant from recombinant HA host strains. Nan (**a**), Pex (**b**) and Ran-H (**c**) were detected using an anti-6x-His-tag monoclonal antibody. Ran-L (**c**) was detected using an anti-FLAG-tag monoclonal antibody. Nan and Pex were analyzed under reducing condition. Ran was analyzed under reducing (R, left lane in each panel) and non-reducing (NR, right lane of each panel) conditions. Arrows indicate correctly assembled proteins. MW, protein ladder.


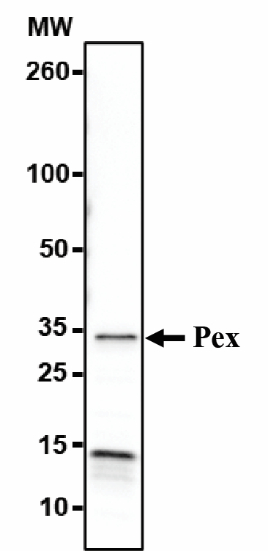


**Figure S2** Expression of Pex after R130K codon mutation. Cell supernatant was collected from the cultivation of the HA.Pex(R130K) strain and analyzed by western blot using the anti-6x-His-tag monoclonal antibody. MW, protein ladder.


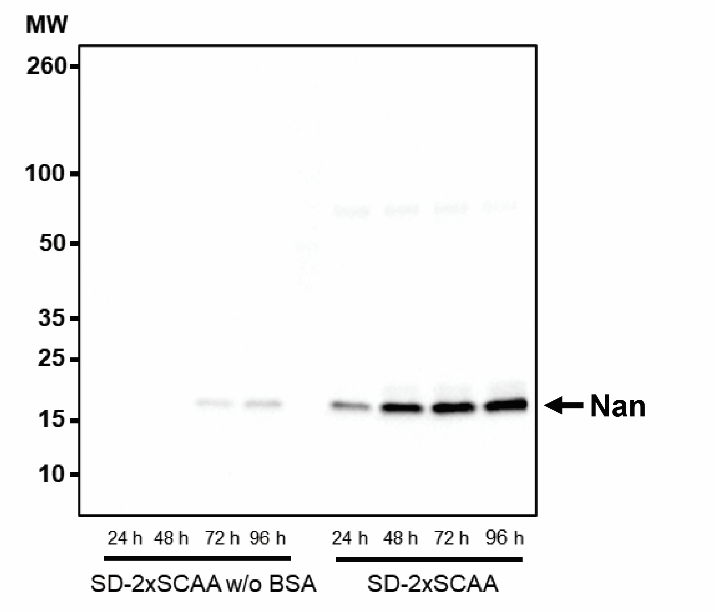


**Figure S3** Influence of BSA in the medium on protein production. Cell supernatants were collected from the cultivations of LA.Nan in SD-2xSCAA medium and SD-2xSCAA without BSA after 24 h, 48 h, 72 h and 96 h. The diluted supernatants were analyzed by western blot using anti-6x-His-tag monoclonal antibody. MW, protein ladder.


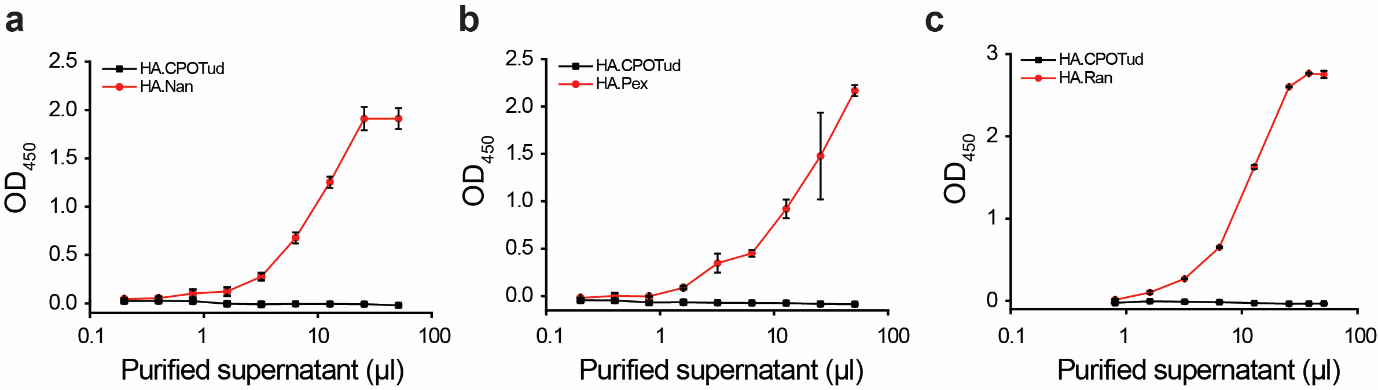


**Figure S4** Binding activity of Nan, Pex and Ran fragments after immunoprecipitation. The binding activity of purified proteins Nan (**a**), Pex (**b**) and Ran (**c**) to the corresponding antigens lysozyme from chicken egg white, complement C5 from human serum and human recombinant VEGF protein, respectively. Supernatants from HA.Nan, HA.Pex, HA.Ran and control HA.CPOTud strains were collected after 72 h of cultivation in SD-2xSCAA medium. The supernatants were immunoprecipitated using Dynabeads™ magnetic bead-based technology. The concentrations of purified proteins from HA.Nan, HA.Ran and HA.Pex were 6.23 ng/mL, 0.61 ng/mL, 0.31 ng/mL, respectively. Three antibody fragments were detected by ELISA signals (absorbance values are displayed as OD_450_) using anti-6x-His-tag monoclonal antibody and HA with the empty plasmid as a negative control. The purified proteins from HA.Nan, HA.Ran and HA.Pex were diluted 60-fold, not diluted or concentrated, and 130-fold concentrated, respectively, when the ELISA assay was performed. Results shown are average values ± SD of biological triplicates (Nan) and duplicates (Pex and Ran).


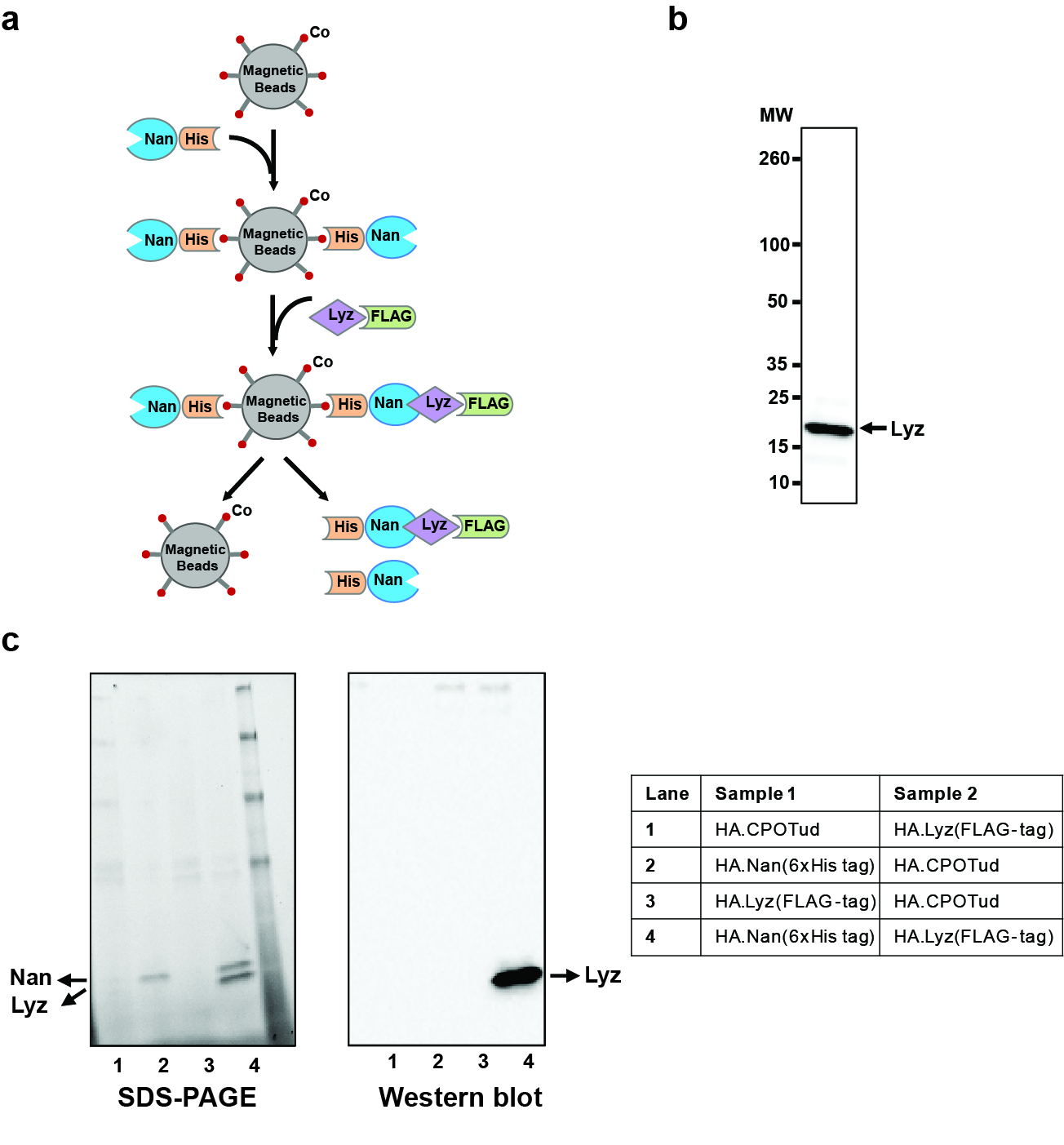


**Figure S5** Pull-down assay for assessing the biological activity. **a** Schematic diagram of the pull-down assay. **b** Western blot using anti-FLAG-tag monoclonal antibody detected the secretion of lysozyme from chicken egg white (Lyz, with FLAG-tag) after the cultivation of HA.Lyz. MW, protein ladder. **c** Pull-down assay between Nan (with 6xHis-tag) and its antigen protein Lyz (with FLAG-tag). Bound proteins were analysed by SDS-PAGE and then probed with an anti-FLAG-tag monoclonal antibody. The sample components are listed in the adjacent table. In the four reactions, Sample 1 was added first and followed by Sample 2, according to the pull-down assay protocol.


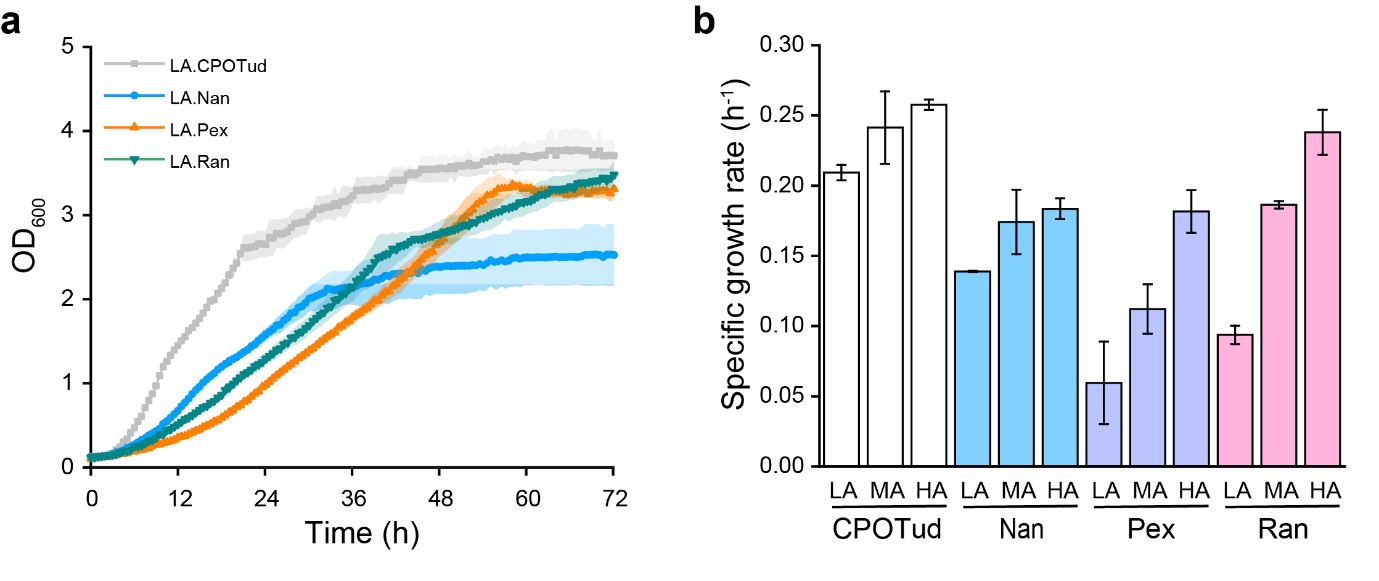


**Figure S6** Growth phenotype of different strains expressing antibody fragments. Cells were grown in microtiter plates with 250 μL SD-2xSCAA medium and 20 g/L glucose as the carbon source. The cell density was monitored by a Growth Profiler. **a** Growth profiles of engineered strains LA.CPOTud, LA.Nan, LA.Pex and LA.Ran. **b** The specific growth rates on glucose of each protein-secreting strain. All data represent average values ± SD of biological triplicates or duplicates and error bars show standard deviations.


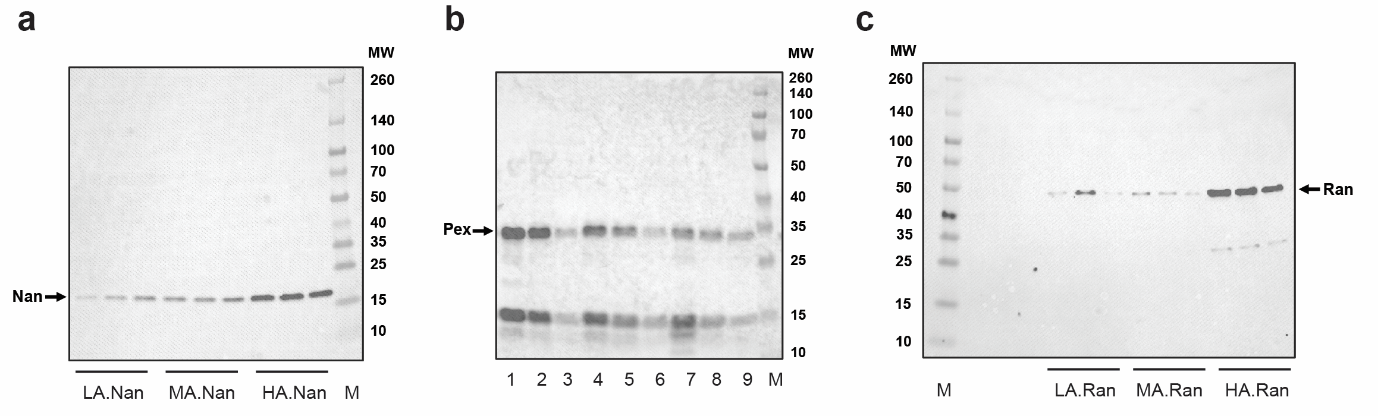


**Figure S7** The original images of the western blots of three different host strains for each protein. All samples for each strain were analyzed in triplicates. Supernatants from the antibody fragment expressing strains were collected after 72 h of cultivation. The western blot using an anti-6x-His-tag antibody was performed under reducing (Nan (**a**) and Pex (**b**)) or non-reducing (Ran (**c**)) conditions. Arrows indicate the expected proteins. M, protein ladder. 1, 4 and 7 represent LA.Pex; 2, 5 and 8 represent MA.Pex; 3, 6 and 9 represent HA.Pex.


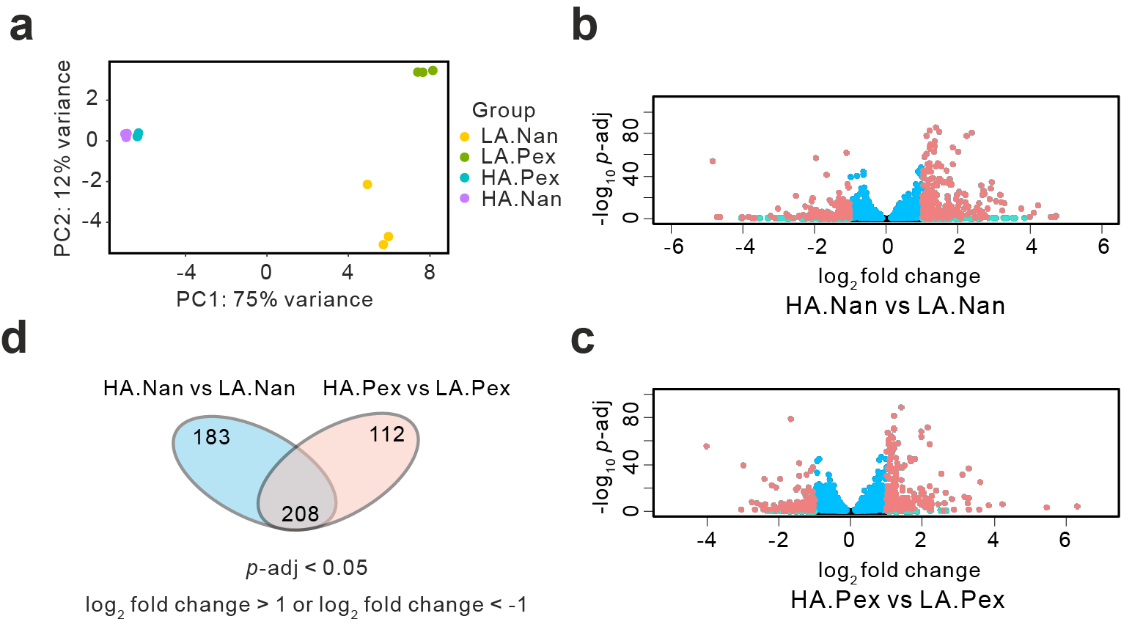


**Figure S8** Global transcriptional response to the expression of Nan and Pex in LA and MA strains. **a** Principal component analysis (PCA) of the normalized RNA-seq data. Samples were taken from biological triplicate cultures. Volcano plot of log_2_ fold change vs adjusted *p*-value (*p*-adj) of differentially expressed genes comparing HA and LA strains expressing protein Nan (HA.Nan vs LA.Nan, **b**) and protein Pex (HA.Pex vs LA.Pex, **c**). *p*-adj was calculated using the Benjamini-Hochberg method. Red circles represent *p*-adj < 0.05 & abs (log_2_ fold change) > 1, blue circles represent *p*-adj < 0.05 & abs (log_2_ fold change) ≤ 1, green circles represent *p*-adj ≥ 0.05 & abs (log_2_ fold change) > 1, black circles represent *p*-adj ≥ 0.05 & abs (log_2_ fold change) ≤ 1. **d** Significantly differentially expressed genes (*p*-adj < 0.05 and abs (log_2_ fold change) > 1) in the mutant strains HA expressing Nan and Pex proteins compared with in LA strains expressing the corresponding proteins.

**
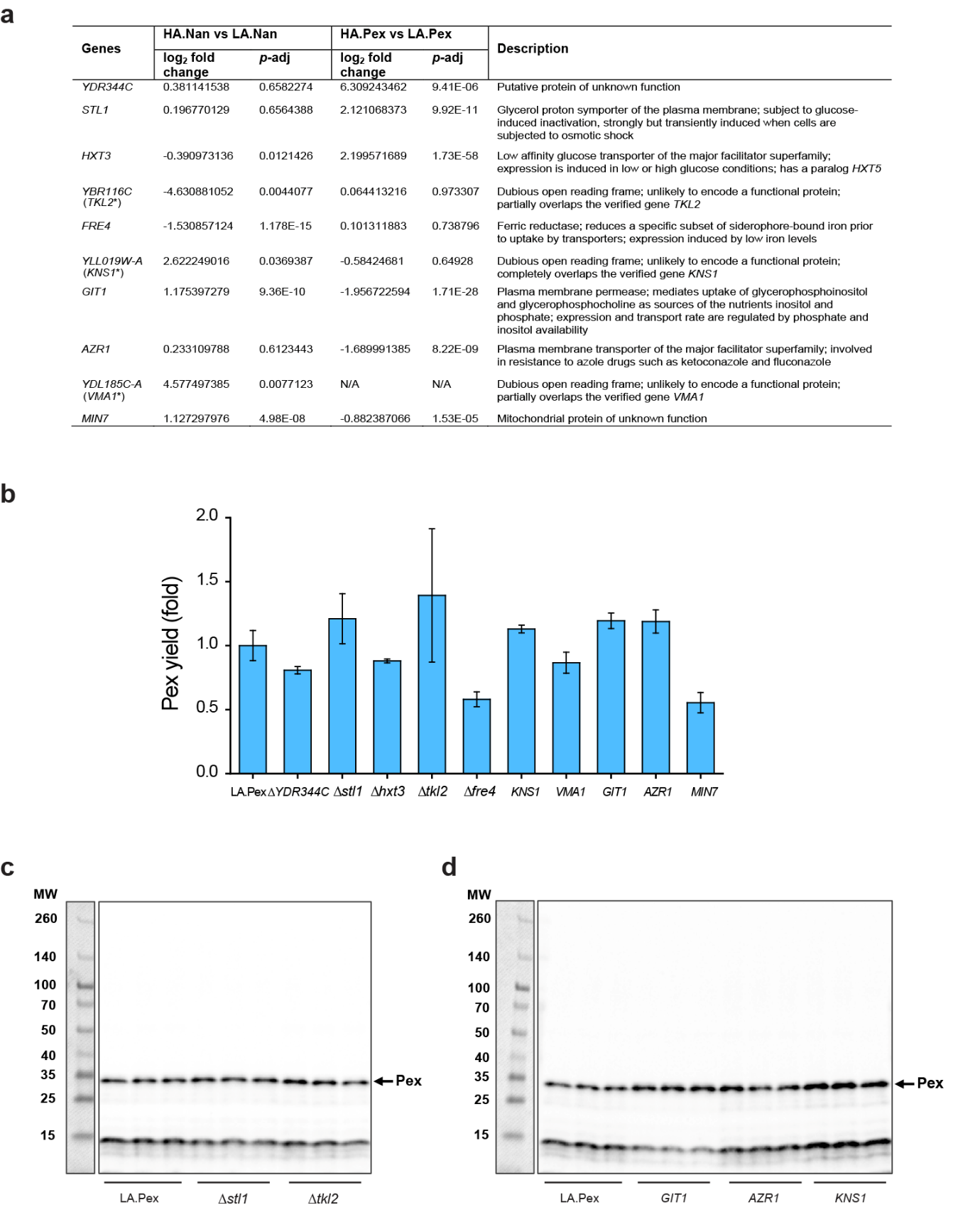
**

**Figure S9** The impact of modified genes on Pex secretion in the LA.Pex strain. **a** The expression values of ten directionally significantly changed genes. Asterisk indicates that the gene partially or completely overlapped with the ORF of the directionally significantly changed gene. **b** Pex yield of strains with single gene deletion or overexpression determined by ELISA signals. Strains were cultivated in a SD-2×SCAA medium at 30 °C for 72 h. **c** and **d** Pex yield of strains with single gene deletion or overexpression determined by western blot. Strains were cultivated in SD-2×SCAA without BSA at 30°C for 72 h. MW, protein ladder. Results shown in **b** are average values ± SD of biological triplicates.


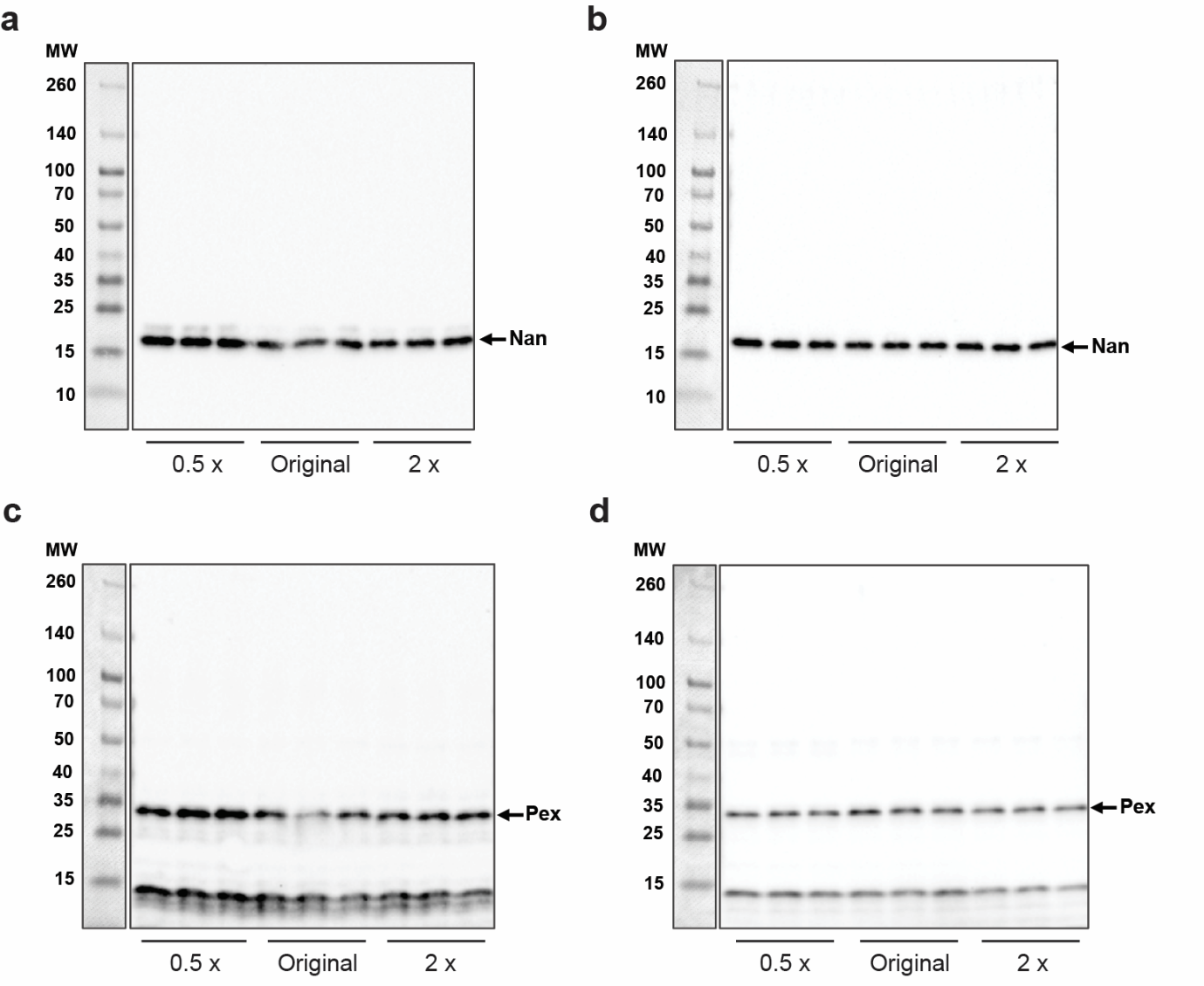


**Figure S10** Effect of changes in glycine concentration on protein production. The concentration of glycine in SD-2xSCAA without BSA medium was 0.5 x the original concentration, original concentration and 2 x the original concentration, respectively. Western blots of cell supernatant from recombinant strains LA.Nan (**a**), HA.Nan (**b**), LA.Pex (**c**) and HA.Pex (**d**) were performed to characterize protein production. MW, protein ladder.


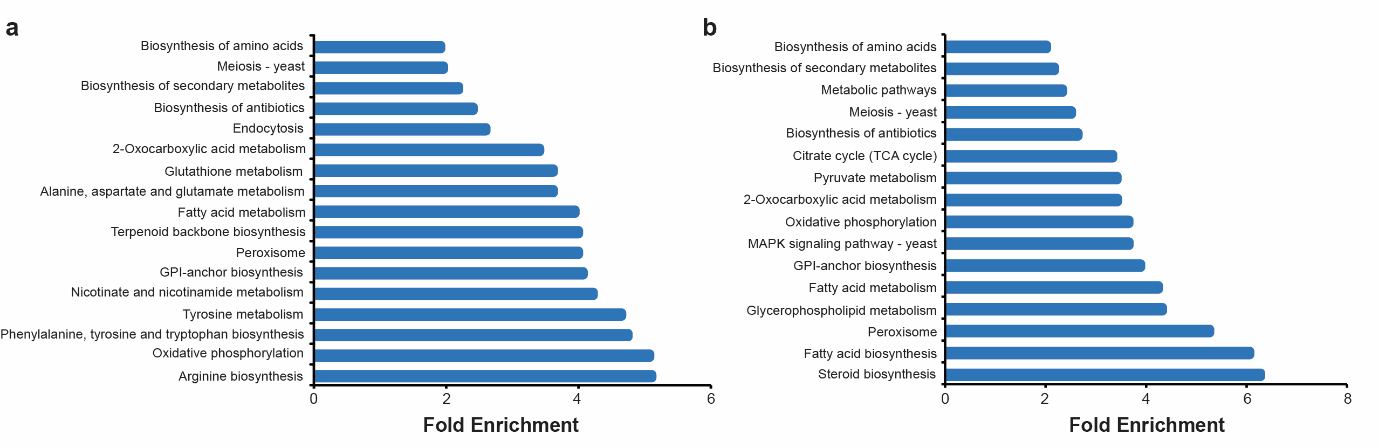


**Figure S11** Gene set enrichment analysis. The gene set enrichment analysis was performed against the S288C yeast genome background using DAVID bioinformatics resources. The down-regulated pathways are shown as HA.Nan vs LA.Nan (**a**) and HA.Pex vs LA.Pex (**b**) (*p*-adj < 0.05 according to the Benjamini–Hochberg method).
