# Supplementary material for: Expression of antibody fragments in Saccharomyces cerevisiae strains evolved for enhanced protein secretion
Source: Microb Cell Fact. 2021 Jul 14;20:134. doi: 10.1186/s12934-021-01624-0 (PMC8278646; doi:10.1186/s12934-021-01624-0)
Supplement: Supplementary file 4 — Additional file 4: Table S1. List of strains and plasmids. Table S2. List of oligonucleotide primer sequences. Table S3. Codon-optimized heterologous genes. [file 12934_2021_1624_MOESM4_ESM.docx]

**Table S1** List of strains and plasmids

| **Plasmids and strains** | **Relevant genotype** | **Origin** |
| --- | --- | --- |
| **Plasmids** | | |
| pUC57-Nan | Codon optimized *alpha factor leader-Nan* gene | GenScript Co. |
| pUC57-Pex | Codon optimized *alpha factor leader-Pex* gene | GenScript Co. |
| pUC57-Ran | Codon optimized *alpha factor leader-Ran* gene | GenScript Co. |
| pUC57-Lyz | Codon optimized *alpha factor leader-Lyz* gene | GenScript Co. |
| CPOTud | 2 μm plasmid with *POT1* gene from *S. pombe* and *TPI1p* and *TPI1t* | [1] |
| pAlphaAmyCPOT | CPOTud with alpha factor leader and α-Amylase gene | [1] |
| pSynInsCPOT | CPOTud with YAP3-TA57 leader and Insulin gene | [1] |
| pUG-amdSYM | AmpR, *TEF2p*-*amdS*-*TEF2t* | [2] |
| pCP-Nan | CPOTud-(*TPI1p-alpha factor leader-Nan gene-TPI1t*) | This study |
| pCP-Pex | CPOTud-(*TPI1p-alpha factor leader-Pex gene-TPI1t*) | This study |
| pCP-Ran | CPOTud-(*TPI1p-alpha factor leader-Ran gene-TPI1t*) | This study |
| pCP-Lyz | CPOTud-(*TPI1p-alpha factor leader-Lyz gene-TPI1t*) | This study |
| pCP-Pex(R130K) | CPOTud-(*TPI1p-alpha factor leader-Pex gene* (R130K)*-TPI1t*) | This study |
| **Strains** |  |  |
| CEN.PK 530-1C | *MATa URA3 HIS3 LEU2 TRP1 SUC2 MAL2-8c*  *tpi1(41-707)::loxP-KanMX4-loxP* | SRD GmbH^a^ |
| AAC | CEN.PK 530-1C with plasmid pAlphaAmyCPOT | [3] |
| M715 | Derived from AAC by UV mutagenesis | [3] |
| MH34 | Derived from M715 by UV mutagenesis | [3] |
| B184 | Derived from MH34 by UV mutagenesis | [3] |
| LA | Eliminated amylase expression plasmid pAlphaAmyCPOT from AAC | This study |
| MA | Eliminated amylase expression plasmid EAlphaAmyCPOT from mutant strain MH34 | This study |
| HA | Eliminated amylase expression plasmid pAlphaAmyCPOT from mutant strain B184 | This study |
| HA.CPOTud | HA with CPOTud | This study |
| HA.Nan | HA with pCP-Nan | This study |
| HA.Pex | HA with pCP-Pex | This study |
| HA.Ran | HA with pCP-Ran | This study |
| HA.Lyz | HA with pCP-Lyz | This study |
| HA.Pex(R130K) | HA with pCP-Pex(R130K) | This study |
| LA.CPOTud | LA with CPOTud | This study |
| LA.Nan | LA with pCP-Nan | This study |
| LA.Pex | LA with pCP-Pex | This study |
| LA.Ran | LA with pCP-Ran | This study |
| MA.CPOTud | MA with CPOTud | This study |
| MA.Nan | MA with pCP-Nan | This study |
| MA.Pex | MA with pCP-Pex | This study |
| MA.Ran | MA with pCP-Ran | This study |
| LA.Pex *∆YDR344C* | LA.Pex *ΔYDR344C::amdS* | This study |
| LA.Pex *∆stl1* | LA.Pex *Δstl1::amdS* | This study |
| LA.Pex *∆hxt3* | LA.Pex *Δhxt3::amdS* | This study |
| LA.Pex *∆tkl2* | LA.Pex *Δtkl2::amdS* | This study |
| LA.Pex *∆fre4* | LA.Pex *Δfre4::amdS* | This study |
| LA.Pex *KNS1* | LA.Pex *amdS-TEF1p-KNS1* | This study |
| LA.Pex *GIT1* | LA.Pex *amdS-TEF1p-GIT1* | This study |
| LA.Pex *AZR1* | LA.Pex *amdS-TEF1p-AZR1* | This study |
| LA.Pex *VMA1* | LA.Pex *amdS-TEF1p-VMA1* | This study |
| LA.Pex *MIN7* | LA.Pex *amdS-TEF1p-MIN7* | This study |

^a^ Scientific Research and Development GmbH, Oberursel, Germany.

**Table S2** List of oligonucleotide primer sequences

| **Primers** | **Sequences (5’-3’)** |
| --- | --- |
| **Construction of plasmids** | |
| TvF | ACTGACAGGTGGTTTGTTACGC |
| TvR | ATCACTTGTAAATCTACCGTCCCT |
| TPexm | CCAGTTCTTTTAATTTCAACCTTTG |
| TRanm | AACTCTGTCACCAACTGAAGCAG |
| TLyzm | TTCTACCATCATTACACCACCATC |
| kex2F  kex2R | CAAAGGTTGAAATTAAAAAAACTGGTGGTGG  CCACCACCAGTTTTTTTAATTTCAACCTTTG |
| **Strain verification** | |
| tPFAF | ATTACTCGGAAGCAAACAAGTTGAGG |
| tPFAR | TTGGCAGGGAACGCAACATC |
| tDOAF | CTCCCAGTATAAGACATTCAATGGAC |
| tDOAR | CAGAATACCTTTCGATCCCAACTT |
| **Deletion and promoter replacement cassette construction** | |
| pYDR344C-F | CTTCACTCCATTTATTACTGG |
| pYDR344C-amdS-R | caagactgtcaaggagggtattctgggcctccatgtcGTGGTTGGACTTCTTTTGAGTTTTC |
| amdS-F | gacatggaggcccagaatac |
| tYDR344C-amdS-R | GTGGTTGGACTTCTTTTGAGTTTTCCGTCCGGATTTCAAAcagtatagcgaccagcattc |
| tYDR344C-F | TTTGAAATCCGGACGGAAAACTCAAAAGAAGTCCAACCACGTGACAACGAGCACGGGTGC |
| tYDR344C-R | GTGTAGATAAAACCGAGTGGG |
| pSTL1-F | GGCACCATCCGTTTCATTTTC |
| pSTL1-amdS-R | caagactgtcaaggagggtattctgggcctccatgtcGTATGCTAGAACACGTATACAAAATAATTATCC |
| tSTL1-amdS-R | GTATGCTAGAACACGTATACAAAATAATTATCCAAAATCAcagtatagcgaccagcattc |
| tSTL1-F | TGATTTTGGATAATTATTTTGTATACGTGTTCTAGCATACCTTAAGAAAGCATTTTGCGGGG |
| tSTL1-R | GTGATGTATGGTGTCCCTTGC |
| pHXT3-F | CTATTCGTCATCGCAGACAG |
| pHXT3-amdS-R | caagactgtcaaggagggtattctgggcctccatgtcAGCTATTAAGTAAAATTATGGAATATTCATGC |
| tHXT3-amdS-R | AGCTATTAAGTAAAATTATGGAATATTCATGCTGAAGATAcagtatagcgaccagcattc |
| tHXT3-F | TATCTTCAGCATGAATATTCCATAATTTTACTTAATAGCTCCGTAGTGCTGAATAATAGTGT  ATTTTAAG |
| tHXT3-R | GTATTCCGGCTGATAAGCATG |
| pTKL2-F | GGAGGTAGTTTGCTGATTGGC |
| pTKL2-amdS-R | caagactgtcaaggagggtattctgggcctccatgtcCAAATCGAAGACTACAGTAAGAGGG |
| tTKL2-amdS-R | CAAATCGAAGACTACAGTAAGAGGGGACAAGCCAATTGAAcagtatagcgaccagcattc |
| tTKL2-F | TTCAATTGGCTTGTCCCCTCTTACTGTAGTCTTCGATTTGGTCTGAAGAAGTAAACAGTTCTTTGC |
| tTKL2-R | CCACGTTGGTTGAATATTGAATC |
| pFRE4-F | GCTTAGGAAGTAAGGTTACTTTGG |
| pFRE4-amdS-R | caagactgtcaaggagggtattctgggcctccatgtcCTGAGGAGACACGATTAACTGG |
| tFRE4-amdS-R | CTGAGGAGACACGATTAACTGGAGTCTTTCTCAGCTTCTCcagtatagcgaccagcattc |
| tFRE4-F | GAGAAGCTGAGAAAGACTCCAGTTAATCGTGTCTCCTCAGACTCAATGTATAATATCCTCTAACT GTTG |
| tFRE4-R | CATGATGAACCAAATGTCTTGTAG |
| pKNS1-F | GCTAGACGCTGTTGGTAATATC |
| pKNS1-amdS-R | catgcgtcaatcgtatgtgaatgctggtcgctatactgTAGATTAGTCTTTGTTGTGCCAAATC |
| amdSOE-F | cagtatagcgaccagcattc |
| amdS-KNS1-R | TAGATTAGTCTTTGTTGTGCCAAATCTTCGACTTGGTAAATACgacatggaggcccagaatac |
| pTEF1-KNS1-F | GTATTTACCAAGTCGAAGATTTGGCACAACAAAGACTAATCTAATAGCTTCAAAATGTTTCTACT  CCTT |
| pTEF1-R | TTTGTAATTAAAACTTAGATTAGATTGCTATGC |
| KNS1-F | GAAAGCATAGCAATCTAATCTAAGTTTTAATTACAAAATGTCGCAGAATATTCAAATTGG |
| KNS1-R | CTATCCTTGGGTATTATTATAAGTTGCA |
| pGIT1-F | GAGCATGACGATGAGTACTTAC |
| pGIT1-amdS-R | catgcgtcaatcgtatgtgaatgctggtcgctatactgCTGCGGCTGGTTGACTAGTC |
| amdS-GIT1-R | CTGCGGCTGGTTGACTAGTCACAAGAAACAGATAATAAATACgacatggaggcccagaatac |
| pTEF1-GIT1-F | GTATTTATTATCTGTTTCTTGTGACTAGTCAACCAGCCGCAGATAGCTTCAAAATGTTTCTACTCCTT |
| GIT1-F | GAAAGCATAGCAATCTAATCTAAGTTTTAATTACAAAATGGAAGATAAAGATATCACATCGG |
| GIT1-R | TCAACTTTGATCGACCTGTCTG |
| pAZR1-F | CGCAGACTCTTTAAATGTTTTCG |
| pAZR1-amdS-R | catgcgtcaatcgtatgtgaatgctggtcgctatactgAGAAAAGATACAAGATTTAAAGGACG |
| amdS-AZR1-R | AGAAAAGATACAAGATTTAAAGGACGTTGCAAGAGGGTACgacatggaggcccagaatac |
| pTEF1-AZR1-F | GTACCCTCTTGCAACGTCCTTTAAATCTTGTATCTTTTCTATAGCTTCAAAATGTTTCTACTCCTT |
| AZR1-F | GAAAGCATAGCAATCTAATCTAAGTTTTAATTACAAAATGAAAGGCGAACCTAAGAC |
| AZR1-R | TTATCTCAGGCTTGCTGTGAC |
| pVMA1-F | GCCCAAATGATATGTCAACCAG |
| pVMA1-amdS-R | catgcgtcaatcgtatgtgaatgctggtcgctatactgTCCTGCGTTATAATCTTAGATATCTATATGC |
| amdS-VMA1-R | TCCTGCGTTATAATCTTAGATATCTATATGCTTTGAGAAATGgacatggaggcccagaatac |
| pTEF1-VMA1-F | CATTTCTCAAAGCATATAGATATCTAAGATTATAACGCAGGAATAGCTTCAAAATGTTTCTACTC CTT |
| VMA1-F | GAAAGCATAGCAATCTAATCTAAGTTTTAATTACAAAATGGCTGGTGCAATTGAAAA |
| VMA1-R | GTTTTGGCAACTTGTGGTTC |
| pMIN7-F | GTAGCATTCAAGGGTAGAAGG |
| pMIN7-amdS-R | catgcgtcaatcgtatgtgaatgctggtcgctatactgTGGTTTTCGCCTTGTCACAG |
| pTEF1-MIN7-F | CTTTTATATTCCACATTTATTCCCTGTGACAAGGCGAAAACCAATAGCTTCAAAATGTTTCTACT  CCTT |
| MIN7-F | GAAAGCATAGCAATCTAATCTAAGTTTTAATTACAAAATGCTGGCCATGAAATCATT |
| MIN7-R | GAATTTCATGCCAGGTCACG |
| **Deletion and promoter replacement cassette confirmation** | |
| vpYDR344C-F | CTACTACATGGGCTCCAATTC |
| vamdS-R | cgataacagagtcttcagctg |
| vpSTL1-F | GCGACCTAGAACCACTAATC |
| vpHXT3-F | GCGTCCAATTTCAACCTAAGG |
| vpTKL2-F | GGTGAGCCTAAAAAGTTCCC |
| vpFRE4-F | GAATGTTTAGTGCATTGGCTC |
| vpKNS1-F | GATTACTGGCAAAGGACTACC |
| vamdSOE-R | caaggctgtttctgaattgg |
| vamdSOE-F | cgataacagagtcttcagctg |
| vKNS1-R | GTCCACTCGACTACTGTTATG |
| vtKNS1-F | GAAACAGGATTACGGCGATAG |
| vtKNS1-R | GTTGCCAAGTGTACAAAGGAG |
| vpGIT1-F | CGATATCTGCGATAAGGTTCT |
| vGIT1-R | CCAATAACCAAGATAGCAGTGG |
| vtGIT1-F | CTGCTGTGACTGGTAAAATC |
| vtGIT1-R | TCAACATGGTGTTCCAAAGC |
| vpAZR1-F | GCAAAAGATCATCCCAGATG |
| vAZR1-R | GCGTTTGGTAAGCTATACCC |
| vtAZR1-F | GAGGTGTCATCTCTAACACTG |
| vtAZR1-R | CTATTGAAGGTGACATAGAGGC |
| vpVMA1-F | CAAAGAGAGATGAATCCGTGC |
| vVMA1-R | GACAGAGGCTTACCTGTTCT |
| vtVMA1-F | CTGAGAGAGCCAACGAATTAG |
| vtVMA1-R | GCCATTCATGTCAACCTTAGC |
| vpMIN7-F | CTCTGGTCCTTTGCTTGTAT |
| vMIN7-R | CCTGGTGTATGATCGTTGAG |
| vtMIN7-F | GTGGGATTGCAGTTTTAACTG |
| vtMIN7-R | CAGCGCTAATCACTTATCAAC |

# Red nucleotide sequence indicates mutated site; lowercase sequence indicates the sequence in amdS cassette.

**Table S3** Codon-optimized heterologous genes

| **Synthesized genes** | **Sequence (5’-3’)** |
| --- | --- |
| *Nan* | GATGTTCAATTGCAAGCTTCTGGTGGTGGTTCAGTTCAAGCAGGTGGTTCTTTGAGATTATCATGTGCTGCATCTGGTTATACTATTGGTCCATACTGTATGGGTTGGTTTAGACAAGCTCCTGGTAAAGAAAGAGAAGGTGTTGCTGCAATTAATATGGGTGGTGGTATCACATACTACGCTGATTCAGTTAAGGGTAGATTCACTATCTCTCAAGATAACGCTAAAAATACAGTTTATTTGTTGATGAATTCATTGGAACCAGAAGATACTGCTATCTATTACTGTGCTGCAGATTCAACAATCTATGCATCTTACTACGAATGTGGTCATGGTTTATCAACTGGTGGTTATGGTTACGATTCTTGGGGTCAAGGTACTCAAGTTACAGTTTCTTCAGGTAGATATCCATACGATGTTCCAGATTATGGTTCTGGTAGAGCACATCATCACCATCACCATTAAgctagc |
| *Pex* | ATGGATATGAGAGTTCCAGCTCAATTGTTGGGTTTGTTGTTGTTGTGGTTGAGAGGTGCAAGATGTGATATTCAAATGACACAATCACCATCTTCATTATCTGCTTCAGTTGGTGACAGAGTTACTATTACATGTGGTGCTTCTGAAAATATCTATGGTGCATTGAATTGGTATCAAAGAAAACCTGGTAAAGCACCAAAGTTGTTGATCTATGGTGCTACTAATTTGGCAGATGGTGTTCCATCTAGATTTTCTGGTTCAGGTTCTGGTACAGATTTCACTTTGACAATCTCTTCATTGCAACCAGAAGATTTCGCTACTTACTACTGTCAAAACGTTTTGAACACACCATTAACTTTCGGTCAAGGTACAAAGGTTGAAATTAAAAGAACTGGTGGTGGTGGTTCTGGTGGTGGTGGTTCTGGTGGTGGTGGTAGTCAAGTTCAATTGGTTCAATCAGGTGCTGAAGTTAAGAAACCAGGTGCATCAGTTAAAGTTTCTTGTAAGGCTTCAGGTTACATTTTCTCTAACTACTGGATCCAATGGGTTAGACAAGCACCAGGTCAAGGTTTGGAATGGATGGGTGAAATTTTACCAGGTTCAGGTTCTACAGAATACACTGAAAACTTCAAGGATAGAGTTACTATGACAAGAGATACTTCAACATCTACTGTTTACATGGAATTGTCTTCATTGAGATCTGAAGATACAGCTGTTTACTACTGTGCAAGATATTTCTTTGGTTCTTCACCAAATTGGTACTTTGATGTTTGGGGTCAAGGTACATTAGTTACTGTTTCTTCACATCATCACCATCACCATTAAgctagc |
| *Ran-H* | GAAGTTCAATTGGTCGAATCCGGTGGTGGTTTGGTACAACCTGGTGGTTCCTTGAGATTATCCTGCGCTGCCTCCGGTTACGACTTTACTCATTACGGTATGAATTGGGTTAGACAAGCTCCTGGTAAAGGTTTGGAATGGGTTGGTTGGATTAATACTTACACAGGTGAACCAACTTACGCTGCAGATTTCAAGAGAAGATTCACTTTTTCTTTAGATACTTCAAAGTCTACAGCTTATTTGCAAATGAACTCTTTGAGAGCTGAAGATACTGCAGTTTACTACTGTGCAAAGTACCCATACTACTACGGTACATCTCATTGGTACTTTGATGTTTGGGGTCAAGGTACTTTGGTTACAGTTTCTTCAGCTTCAACAAAAGGTCCATCTGTTTTTCCATTAGCACCATCTTCAAAATCAACTTCTGGTGGTACAGCTGCATTGGGTTGTTTAGTTAAAGATTACTTTCCAGAACCAGTTACTGTTTCATGGAATTCTGGTGCTTTGACTTCAGGTGTTCATACATTTCCAGCAGTTTTGCAATCTTCAGGTTTGTACTCTTTGTCTTCAGTTGTTACAGTTCCATCTTCATCTTTGGGTACTCAAACATACATCTGTAATGTTAATCATAAACCATCAAATACTAAGGTAGATAAAAAGGTAGAACCAAAATCCTGCGATAAAACTCACTTGCATCATCACCATCACCATAGAAGAAAGAGAGGTTCCGGTGAAGGTAGAGGTTCCTTATTGACTTGCGGTGACGTCGAAGAAAATCCTGGTCCT |
| *Ran-L* | GATATACAATTAACTCAATCACCATCTTCATTGTCTGCTTCAGTTGGTGACAGAGTTACTATTACATGTTCTGCATCACAAGATATCTCTAACTATTTGAACTGGTATCAACAAAAACCTGGTAAAGCTCCAAAGGTTTTGATCTATTTCACATCTTCATTGCATTCAGGTGTTCCATCTAGATTTTCTGGTTCAGGTTCTGGTACTGATTTCACTTTGACAATCTCTTCATTGCAACCAGAAGATTTCGCAACATACTACTGTCAACAATACTCTACTGTTCCATGGACATTCGGTCAAGGTACTAAGGTTGAAATTAAAAGAACAGTTGCTGCACCATCAGTTTTTATTTTTCCACCATCTGATGAACAATTGAAATCAGGTACTGCTTCTGTTGTTTGTTTGTTGAACAACTTCTATCCAAGAGAAGCTAAGGTTCAATGGAAGGTTGATAACGCATTGCAATCTGGTAATTCTCAAGAATCAGTTACTGAACAAGATTCTAAGGATTCAACATACTCTTTGTCTTCAACTTTGACATTGTCTAAGGCTGATTACGAAAAGCATAAAGTTTACGCATGTGAAGTAACACATCAAGGTTTATCCAGTCCAGTAACAAAGAGTTTTAACAGAGGTGAATGCGACTACAAGGACGACGATGACAAGTAAgctagc |
| *Lyz* | AAGGTATTCGGTAGATGTGAATTGGCCGCCGCCATGAAGAGACACGGTTTGGACAACTACAGAGGTTACTCCTTGGGTAATTGGGTCTGTGCTGCAAAGTTCGAATCTAACTTCAACACTCAAGCTACAAACAGAAACACTGATGGTTCTACAGATTACGGTATCTTGCAAATTAATTCAAGATGGTGGTGTAATGATGGTAGAACTCCAGGTTCTAGAAATTTGTGTAACATCCCATGTTCAGCATTGTTGTCTTCAGATATCACAGCTTCTGTTAACTGTGCTAAGAAAATTGTTTCAGATGGTAACGGTATGAACGCTTGGGTTGCATGGAGAAATAGATGTAAAGGTACAGATGTTCAAGCCTGGATTAGAGGTTGTAGATTGGACTATAAAGATGATGACGATAAGTAAgctagc |

# Red sequence indicates 6xHis-tag; purple sequence indicates FLAG-tag; yellow, blue and green sequence indicates Kex2 cleavage site, GSG linker and 2A peptide; lowercase sequence indicates restriction site.

**Supplementary References**

1. Liu Z, Tyo KE, Martínez JL, Petranovic D, Nielsen J. Different expression systems for production of recombinant proteins in *Saccharomyces cerevisiae*. Biotechnol Bioeng. 2012;109:1259-1268.

2. Solis-Escalante D, Kuijpers NG, Bongaerts N, Bolat I, Bosman L, Pronk JT, et al. amdSYM, a new dominant recyclable marker cassette for *Saccharomyces cerevisiae*. FEMS Yeast Res. 2013;13:126-139.

3. Huang M, Bai Y, Sjostrom SL, Hallström BM, Liu Z, Petranovic D,et al. Microfluidic screening and whole-genome sequencing identifies mutations associated with improved protein secretion by yeast. Proc Natl Acad Sci USA. 2015;112:E4689-4696.
